# Immunomodulation as a novel treatment strategy for periodontal disease: a systematic review

**DOI:** 10.1186/s12903-026-08120-0

**Published:** 2026-06-15

**Authors:** Fariad Akbar, Marta Czesnikiewicz-Guzik

**Affiliations:** 1 Newcastle Dental Hospital, Newcastle Upon Tyne, UK; 2 Department of Periodontology and Oral Sciences Research Group, University of Glasgow Dental School, Glasgow, UK; 3 Glasgow Dental Hospital & School, 378 Sauchiehall Street, Glasgow, G2 3JZ UK

**Keywords:** Gingivitis, Immunomodulation, Periodontal Diseases, Periodontal therapy, Periodontitis

## Abstract

**Background:**

Periodontal diseases (PDs) are globally prevalent and are associated with reduced quality of life and systemic health complications. Conventional therapy, involving professional mechanical plaque removal (PMPR) and oral hygiene instruction, faces limitations involving disease recurrence and patient factors. This systematic review explores immunomodulatory therapies as alternative or adjunctive treatments.

**Method:**

MEDLINE (Ovid), EMBASE (Ovid), Cochrane Database of Systematic Reviews and MEDLINE (Web of Science) databases were searched on 9 June 2024. Two authors independently screened the articles using pre-specified inclusion and exclusion criteria, and findings were grouped by immunomodulatory agent.

**Results:**

The search retrieved 2363 records, and 42 were included. Thirteen types of immunomodulatory agents were evaluated across cell lines, animal models of PD, and observational studies and clinical trials in humans. Several agents showed evidence of effects on inflammation and alveolar bone loss; however, the quality and consistency of evidence varied. Limited data suggest that longer treatment duration, alternative delivery methods, or combination approaches may enhance therapeutic outcomes.

**Conclusion:**

Immunomodulatory agents show promise for the management of PD, including in patients with co-morbidities. However, clinical evidence in the context of conventional therapy and safety data remain limited. Further rigorous trials are needed to confirm efficacy, optimise treatment, and evaluate long-term safety before these therapies can be incorporated into routine clinical practice.

**Supplementary Information:**

The online version contains supplementary material available at https://doi.org/10.1186/s12903-026-08120-0.

## Background

Periodontal disease (PD) encompasses a group of inflammatory conditions, commonly gingivitis and periodontitis, which affect the periodontal tissues including the gingivae, periodontal ligament and alveolar bone [1–6]. Untreated or poorly controlled periodontitis risks tooth loss [2, 3, 6–12], indicating the need for effective prevention and treatment to conserve quality of life including masticatory efficiency, dietary choices and self-confidence [4, 13].

The aetiopathogenesis of PD is dependent on dysbiotic changes within dental plaque, a bacterial biofilm [6, 14, 15]. Specifically, plaque colonisation by Gram-negative anaerobes such as *Fusobacterium nucleatum* and *Prevotella* species is associated with the onset of gingivitis [3, 6, 14], whereas, colonisation of mature plaque by *Porphyromonas gingivalis* (*P.g.*), *Treponema denticola* (*T.d.*) and *Tannerella forsythia* (*T.f.*) is associated with the onset of periodontitis [6, 16, 17]. As these anaerobes represent the dysbiotic change required to induce PD, they are termed periodontal pathogens or keystone pathogens [8, 18, 19]. Although dysbiotic plaque is the causal factor, the host immune response is a principal driver of PD-induced periodontal tissue destruction [2, 8, 12, 16, 17, 19–27].

Gingival epithelial cells, the complement cascade, neutrophils and macrophages induce early connective tissue damage by releasing toxic substances into the extracellular matrix, including matrix metalloproteinases (MMPs) and reactive oxygen species [5, 10, 11, 22, 28, 29]. Antigen-specific B and T lymphocytes of the later adaptive immune response contribute to connective tissue damage through the release of MMPs and autoantibodies, and induce alveolar bone destruction through receptor activator of nuclear factor kappa-B ligand (RANKL)- and interleukin (IL)-1β-mediated osteoclast mechanisms [5, 7, 10, 26–30].

The breakdown of self-tissue creates a nutrient source for plaque bacteria. Furthermore, periodontal pathogens have evolved mechanisms to evade or manipulate the host immune response to facilitate their survival [6, 15, 18]. Continued proliferation of plaque anaerobes creates a sustained inflammatory stimulus, leading to chronic inflammation in untreated PD [6, 31]. When endogenous immune regulators (e.g. the T regulatory [Treg] subset of T lymphocyte, and regulatory B lymphocytes) fail to control the inflammation, progressive periodontal tissue destruction ensues [18, 29, 31].

Current conventional therapy for PD entails non-surgical mechanical removal of dental plaque to reduce the bacterial load and support a return to microbial symbiosis [6, 9, 32–34]. Although clinically effective in disrupting plaque biofilms, a major disadvantage is the rapid, spontaneous re-accumulation of dental plaque, indicating frequent, repeated professional mechanical plaque removal (PMPR, previously termed scaling and root planing [SRP]) and oral hygiene instruction [4–6, 9, 23, 35, 36] which introduces economic barriers [4, 26, 37]. Despite compliance, the periodontal tissues of some patients respond inadequately to this therapy [12, 23, 31], while others experience physical barriers including impaired manual dexterity [6, 38, 39]. Considering the principal role of our immune response in mediating periodontal tissue destruction, immune-targeting therapies may be a valuable alternative or adjunctive treatment approach [22, 40, 41]. Such therapies can be sub-divided into immunosuppressive and immunomodulatory therapies [42].

Immunosuppressive therapies, including corticosteroids and non-steroidal anti-inflammatory drugs, have a broader immune-dampening effect and disrupt the immune response at multiple stages [42]. Trials investigating their adjunctive use with PMPR were inconclusive [43–45], with evidence suggesting such global immune suppression has unintended consequences on protective antimicrobial elements of the immune response as well as linked physiological mechanisms [6, 43, 44, 46]. As immunomodulatory therapies more selectively target immune mechanisms, they can theoretically avoid these complications and thus minimise side effects while enhancing the efficacy of treatment [4, 23, 42].

This systematic review aims to evaluate whether immunomodulatory therapies have the potential, either as standalone or adjuncts to conventional periodontal therapy, to improve periodontal outcomes.

## Methods

This review conforms to the PRISMA 2020 checklist – see Supplementary file 1 [47]. The protocol for this review was not pre-registered.

Clinical trial number: not applicable.

### Research aim

The research aim was developed using the PICO framework – see Table 1. In brief, this systematic review aimed to explore whether immunomodulatory therapies tested in cell/tissue cultures, animals or humans indicated measurable outcomes for treatment of PD. Therapies were defined as either standalone or adjuncts to conventional periodontal therapy, and outcomes of interest included clinical periodontal parameters or inflammatory changes associated with PD.

**Table 1:** Description of PICO outline for research aim

Population	Animals or patients with gingivitis or periodontitis, or Cell or tissue cultures exposed to periodontal pathogens or immune mediators involved in periodontal inflammation
Intervention	Immunomodulatory therapy alone, or Conventional periodontal therapy (i.e. professional mechanical plaque removal with or without oral hygiene instruction) with adjunctive immunomodulatory therapy
Comparison	No periodontal therapy, or Conventional periodontal therapy alone
Outcome	Reduction in gingival bleeding, or Changes in periodontal/probing pocket depth, or Changes in clinical attachment loss, or Changes in alveolar bone levels/loss, or Changes in cytokine levels in blood or tissue, or Changes in immune cell count in blood or tissue, or Reduction in number of teeth lost due to periodontal disease

### Study selection

The inclusion and exclusion criteria (see Table 2) were developed in line with the research aim, and discussions between the authors were guided by the PICO framework (Table 1). Briefly, only articles published in English were considered for inclusion; this language restriction was applied as a filter during the database searches. Eligible studies were published in high-impact journals and investigated immunomodulatory agents without any antibacterial, antimicrobial or bactericidal properties to ensure that observed effects on periodontal outcomes could be attributed specifically to immunomodulation.

**Table 2:** Inclusion/exclusion criteria

Inclusion criteria	Exclusion criteria
• Published in English (language restriction applied at the search stage) • Randomised controlled trials (RCTs), observational studies, animal studies, in vitro studies o Only in vitro studies exclusively investigating immunomodulatory properties of a (potential) therapeutic agent o Only clinical, observational and animal studies investigating a therapeutic agent with known immunomodulatory properties • Reports published in a ‘high-impact’ journal, defined as a Journal Impact Factor (JIF) within the top first quartile (Q1) for all applicable categories on Clarivate Journal Citation Reports or Scimago Journal & Country Rank	• Reports which do not relate to the research question. This includes reports investigating probiotic therapy, antibiotic/antimicrobial/antiviral therapy, phototherapy, regenerative therapy, or dietary modification/supplementation (all outwith the scope of this review) • Reports with inconsistent results/findings • Meta-analyses, systematic reviews and literature reviews; primary data sources were added to inclusion list if they fulfilled the inclusion criteria • Reports investigating an immunosuppressive agent which impairs the immune response through multiple mechanisms (e.g. corticosteroids and non-steroidal anti-inflammatory drugs) • Reports investigating a therapeutic agent with reported antibacterial/antimicrobial/bactericidal properties in addition to immunomodulatory properties, since it will be unclear which of these properties causes any recorded improvement in clinical periodontal outcomes • Reports published in a low-impact journal • Inaccessible papers (unable to access through Open Access or University of Glasgow credentials)

### Search strategy

The following electronic databases were manually searched by both authors on 9 June 2024: MEDLINE (Ovid), EMBASE (Ovid), Cochrane Database of Systematic Reviews, and MEDLINE (Web of Science). The search strategy for each database (see Table 3) included synonyms combined with the Boolean operator “OR” to achieve sensitivity within concepts. The concepts were combined with the Boolean operator “AND” to ensure that each concept was represented in the final search.

**Table 3:** Search strategy for selected databases

Database	Search strategy
*Medline (Ovid)*	**#1** immunomodulat*.ti, ab; **#2** (immune adj3 (modulat* OR therap*)).ti, ab; **#3** immunotherap*.ti, ab; **#4** (biological adj3 therap*).ti, ab; **#5** *immunomodulation/; **#6** periodontitis.ti, ab; **#7** (periodontal adj2 disease*).ti, ab; **#8** gingivitis.ti, ab; **#9** (gingival adj2 disease*).ti, ab; **#10** *periodontal diseases/; **#11** *gingival diseases/; **#12** *periodontitis/; **#13** *tooth loss/; **#14** *tooth mobility/; **#15** #1 OR #2 OR #3 OR #4 OR #5; **#16** #6 OR #7 OR #8 OR #9 OR #10 OR #11 OR #12 OR #13 OR #14; **#17** #15 AND #16
*EMBASE (Ovid)*	**#1** immunomodulat*.ti, ab; **#2** (immune adj3 (modulat* OR therap*)).ti, ab; **#3** immunotherap*.ti, ab; **#4** (biological adj3 therap*).ti, ab; **#5** *immunomodulation/; **#6** periodontitis.ti, ab; **#7** (periodontal adj2 disease*).ti, ab; **#8** gingivitis.ti, ab; **#9** (gingival adj2 disease*).ti, ab; **#10** *periodontal disease/; **#11** *gingiva disease/; **#12** *periodontitis/; **#13** #1 OR #2 OR #3 OR #4 OR #5; **#14** #6 OR #7 OR #8 OR #9 OR #10 OR #11 OR #12; **#15** #13 AND #14
*Cochrane Database of Systematic Reviews*	**#1** (immunomodulat*):ti, ab, kw; **#2** (immune near/3 (modulat* OR therap*)):ti, ab, kw; **#3** (immunotherap*):ti, ab, kw; **#4** (biological near/3 therap*):ti, ab, kw; **#5** MeSH descriptor: [Immunomodulation] in all MeSH products^a^; **#6** (periodontitis): ti, ab, kw; **#7** (periodontal near/2 disease*):ti, ab, kw; **#8** (gingivitis): ti, ab, kw; **#9** (gingival near/2 disease*):ti, ab, kw; **#10** MeSH descriptor: [Periodontal diseases] this term only; **#11** MeSH descriptor: [Gingival diseases] this term only; **#12** MeSH descriptor: [Periodontitis] in all MeSH products^a^; **#13** MeSH descriptor: [Tooth Loss] this term only; **#14** MeSH descriptor: [Tooth Mobility] this term only; **#15** #1 OR #2 OR #3 OR #4 OR #5; **#16** #6 OR #7 OR #8 OR #9 OR #10 OR #11 OR #12 OR #13 OR #14; **#17** #15 AND #16
*MEDLINE (Web of Science)*	**#1** TI=(immunomodulat*); **#2** AB=(immunomodulat*); **#3** TI=(immune NEAR/3 (modulat* OR therap*)); **#4** AB=(immune NEAR/3 (modulat* OR therap*)); **#5** TI=(immunotherap*); **#6** AB=(immunotherap*); **#7** TI=(biological NEAR/3 therap*); **#8** AB=(biological NEAR/3 therap*); **#9** MH=(Immunomodulation); **#10** TI=(periodontitis); **#11** AB=(periodontitis); **#12** TI=(periodontal NEAR/2 disease*); **#13** AB=(periodontal NEAR/2 disease*); **#14** TI=(gingivitis); **#15** AB=(gingivitis); **#16** TI=(gingival NEAR/2 disease*); **#17** AB=(gingival NEAR/2 disease*); **#18** MH=(Periodontal Diseases); **#19** MH=(Gingival Diseases); **#20** MH=(Periodontitis); **#21** MH=(Tooth Loss); **#22** MH=(Tooth Mobility); **#23** #1 OR #2 OR #3 OR #4 OR #5 OR #6 OR #7 OR #8 OR #9; **#24** #10 OR #11 OR #12 OR #13 OR #14 OR #15 OR #16 OR #17 OR #18 OR #19 OR #20 OR #21 OR #22; **#25** #23 AND #24

^a^ The Cochrane Database of Systematic Reviews does not allow unexploded searching of these MeSH terms; they were therefore exploded for this search only

### Data abstraction

The records identified were exported to EndNote (version X9.3.3) and independently screened by both authors based on titles and abstracts. Full texts of remaining articles were obtained and reviewed, with any uncertainties resolved through discussion until consensus was reached.

The final list of included articles was categorised by immunomodulatory agent type. These categories were developed iteratively by identifying recurring agent classes. This approach allowed studies with comparable mechanisms or targets to be synthesised together in the results. In line with the research aim (Table 1), the authors collaborated to extract all relevant data from each article, including study design, duration, demographics, recruitment protocols, sample size, interventions, controls, outcomes of interest, and significant findings. A standardised approach was used to capture study limitations and potential biases related to study design, methodology, funding sources, and author affiliations. A formal assessment of certainty of evidence was not conducted due to the substantial heterogeneity of study designs, outcomes, and measurements.

## Results

**Fig. 1:**
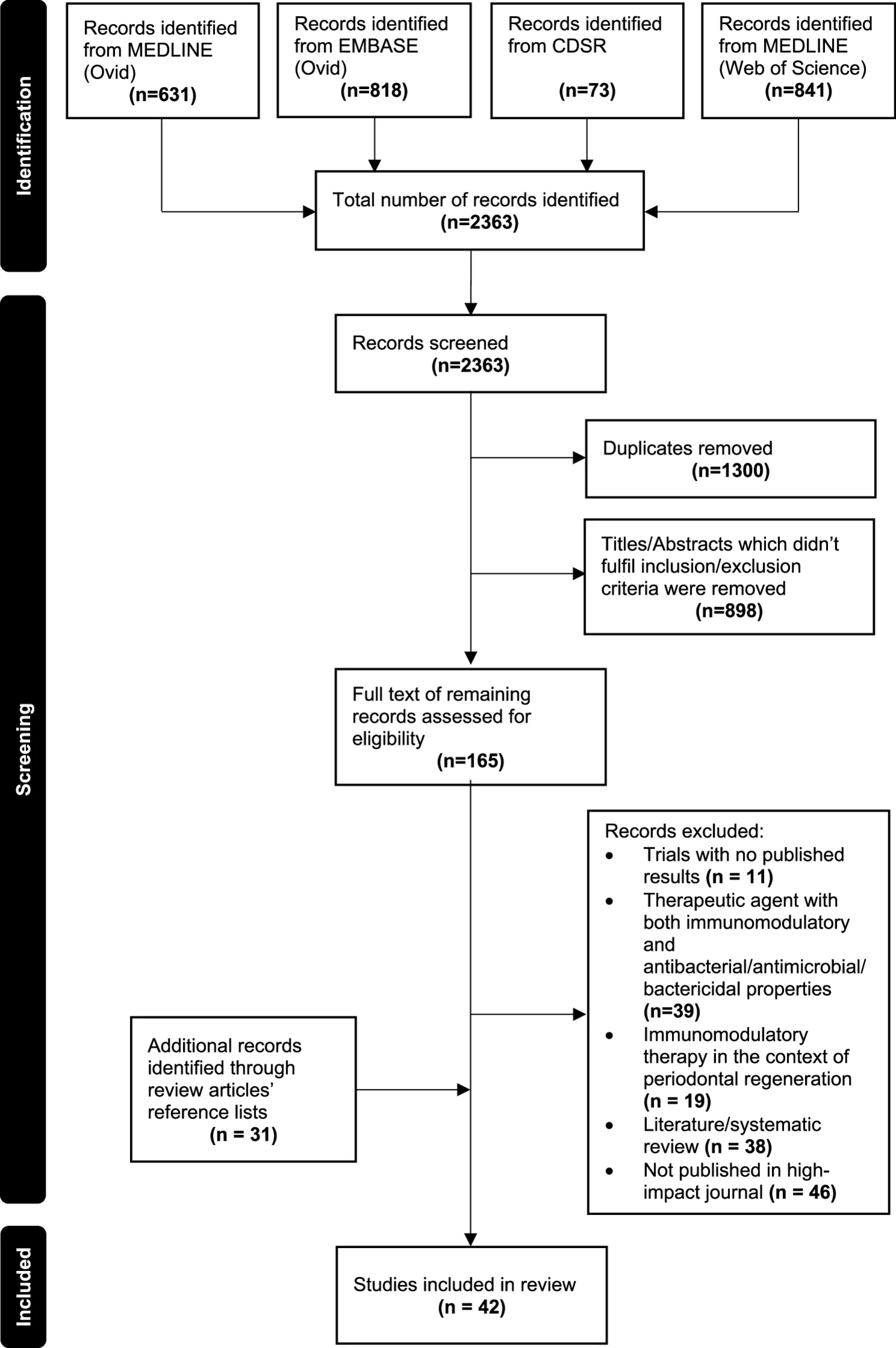
PRISMA 2020 flow diagram for article selection. Adapted from Page et al. (2021)

The literature search was conducted across all databases on 9 June 2024 and yielded 2363 results, of which 1300 were duplicates. A further 1052 results were excluded based on the inclusion/exclusion criteria (Table 2). Manual screening of 38 meta-analyses and systematic/literature reviews identified 31 additional primary articles for inclusion, bringing the total number of included articles to 42. Figure 1 summarises the study selection process as a PRISMA flow diagram [47].

The 42 included articles reported 58 distinct studies; six observational studies and 14 clinical trials, 28 in vivo animal studies, and 10 in vitro experimental studies. The 58 studies were categorised by 13 types of immunomodulatory agents: selective pharmacological inhibitors (*n* = 12 reports) [20, 21, 38, 48–55], tetracyclines at sub-antimicrobial doses (*n* = 12, all investigating doxycycline) [22, 28, 32, 33, 56–62], immunisation agents (*n* = 7) [17, 63], plant-derived compounds (*n* = 6) [1, 2, 7, 31], anti-hyperglycaemic drugs (*n* = 4) [3, 64], vitamins or their derivatives/metabolites (*n* = 3) [16, 65, 66], a chemokine-based agent (*n* = 3) [23], a polymer-loaded nanofibre (*n* = 3) [11], tricyclic antidepressants (*n* = 2) [41, 67], a microRNA-based agent (*n* = 2) [68], a beta-glucan (*n* = 1) [69], strontium ranelate (*n* = 1) [8], and a growth factor (*n* = 1) [12]. One additional study investigated a combination of clodronate and chemically modified non-antimicrobial doxycycline (CMT-8) [24]. Table 4 lists the specific immunomodulatory agents investigated, grouped by category. Table S1 in Supplementary file 2 summarises the included studies and experiments, including their significant findings.

**Table 4:** Summary of immunomodulatory agents investigated for periodontal disease treatment

Type of agent	Immunomodulatory agent investigated	Study count
Tetracyclines at sub-antimicrobial doses	Sub-antimicrobial dose doxycycline (SDD) [22, 28, 32, 56, 57, 59, 61, 62]; Low-dose doxycycline [33, 58, 60]	**12**
Selective pharmacological inhibitors	Infliximab (an anti-TNF-α antibody) [38, 54, 55]; Fluoxetine (a selective serotonin reuptake inhibitor [SSRI]) [48]; Rituximab (an anti-CD20 monoclonal antibody) [49]; Etanercept (a TNF-α inhibitor) [50]; Odanacatib (a cathepsin-K inhibitor) [51]; Tocilizumab (an IL-6 receptor [IL-6R] inhibitor) [21, 52]; Adalimumab (a humanised anti-TNF-α monoclonal antibody) [53]; 1-trifluoromethoxyphenyl-3-(1-propionylpiperidin-4-yl) urea [TPPU] (a soluble epoxide hydrolase inhibitor) [20]	**12**
Immunisation agents	KAS1-sA1 [17]; KAS2-A1; KgpA1(759–989) [17]; KgpA1(751–1056) [17]; Formalin-killed *Porphyromonas gingivalis* (*P.g*.) W50 cells [17]; RgpA-Kgp complex [17]; Anti-*P.g.* egg yolk antibody against gingipains (IgY-GP) [63]	**7**
Plant-derived compounds	Cannabigerol [1]; Cannabidiol [1]; Cannabidivarin [1]; 6-shogaol (a bioactive compound in dried ginger) [2]; CsinCPI-2 (a cysteine peptidase inhibitor derived from Citrus sinensis orange tree) [7]; Boldine (bioactive compound extracted from the Peumus boldus Molina species of boldo tree) [31]	**6**
Anti-hyperglycaemic drugs	Glyburide [3]; Metformin [3, 64]; MCC950 (an NLRP3 inhibitor) [3]; z-YVAD-FMK (a capase-1 inhibitor) [3]; Tolbutamide [3]; Gliclazide [3]; Glimepiride [3]	**4**
Vitamins, their derivates and/or metabolites	L-ascorbic acid 2-phosphate magnesium salt (APM) (a derivative of vitamin C)^a^ [65]; Fenretinide (a synthetic analogue of vitamin A) [66]; All-trans retinoic acid (ATRA, an active metabolite of vitamin A) [16]	**3**
Chemokine-based agents	Recombinant mouse C-C motif chemokine ligand 22 (CCL22) [23]	**3**
Polymer-loaded nanofibres	Poly(ɛ-caprolactone) nanofibres [11]	**3**
Tricyclic antidepressants	Desipramine [41]; Tianeptine [67]	**2**
microRNA-based agents	C-X-C chemokine receptor type 4 (CXCR4)-overexpressing exosome loaded with miR-126 (a macrophage-associated anti-inflammatory microRNA molecule) [CXCR4-miR126-Exo] [68]	**2**
Beta-glucans	Purified beta-1,3/1,6-glucan [β-1,3/1,6-glucan] (a carbohydrate polymer isolated from the cell wall of Saccharomyces cerevisiae) [69]	**1**
Strontium ranelate	Strontium ranelate [8]	**1**
Growth factors	Progranulin [12]	**1**
Combination of agents (co-administration of 2 agents)	6a-deoxy,5 hydroxy-4-dedimethylaminotetracycline [abbreviated as chemically modified tetracycline (CMT)-8] (a chemically modified non-antimicrobial doxycycline); Clodronate (a bisphosphonate) [24]	**1**

^a^ AsANa used as a control (described as equivalent to standard vitamin C)

Ten in vitro experimental studies investigated plant-derived compounds [1, 2, 7], anti-hyperglycaemic drugs [3, 64], vitamins, their derivatives and/or metabolites [65, 66], a polymer-loaded nanofibre (poly(ɛ-caprolactone)-loaded nanofibre with highly-oriented periodic lamellae structure [PLN]) [11], and a microRNA-based agent (C-X-C chemokine receptor type 4 (CXCR4)-overexpressing, microRNA-126-loaded exosome [CXCR4-miR126-Exo]) [68]. All studies demonstrated anti-inflammatory potential. Five of these agent types were also tested in eight in vivo studies, with similar reductions in inflammation observed alongside reduced alveolar bone resorption. These included: three in vivo studies for the plant-derived compounds, boldine, 6-shogaol, and cystatin-like protein of sweet orange (CsinCPI-2) [2, 7, 31]; two for the anti-hyperglycaemic drugs, metformin and glyburide [3, 64]; and one for each of all-trans retinoic acid (ATRA) [16], PLN [11], and CXCR4-miR126-Exo [68].

The combination therapy of CMT-8 and clodronate [24] and the following types of agents were exclusively investigated in vivo: strontium ranelate [8], a beta-glucan (purified beta-1,3/1,6-glucan) [69], a chemokine-based agent (recombinant mouse C-C motif chemokine ligand 22 [CCL22]) [23], tricyclic antidepressants (desipramine [41] and tianeptine [67]), and a growth factor (progranulin-loaded gelatin methacryloyl [PGRN-GelMA]) [12]. CCL22 and the combination therapy both reduced inflammation and bone loss [23, 24]. Beta-1,3/1,6-glucan had a limited anti-inflammatory effect [69], while strontium ranelate was effective only at the highest concentration tested [8]. Desipramine reduced inflammation in control animals with periodontitis [41], while tianeptine showed mixed results, with most serum markers indicating reduced inflammation, except for tumour necrosis factor-alpha (TNF-α), a pro-inflammatory cytokine, which remained elevated [67]. Reduced bone loss was evidenced across all these in vivo studies, except that of PGRN-GelMA which did not assess bone loss but instead focused specifically on bone regeneration. In this context, PGRN-GelMA, as an adjunct to PMPR, reduced inflammation and promoted bone regeneration [12].

The evidence for selective pharmacological inhibitors included five in vivo studies [20, 21, 48, 50, 51] and seven human studies involving immunomodulatory drugs which are clinically prescribed for rheumatoid arthritis (RA); five observational studies [38, 49, 54, 55] and two clinical trials [52, 53].

Infliximab, a TNF-α inhibitor commonly prescribed for RA, was investigated in four human studies. All reported reductions in attachment loss (AL) [38, 54, 55]; two also observed reductions in probing pocket depth (PPD) and improvements in gingival inflammation, as indicated by lower gingival index (GI) scores and reduced percentage sites with bleeding on probing (%BOP) [54, 55]. In contrast, one study and its follow-up reported no improvement in PPD and worsening gingivitis, as indicated by higher modified GI (MGI) and papillary bleeding index scores [38].

An uncontrolled clinical trial of adalimumab, another TNF-α inhibitor approved for RA and Crohn’s disease, involving 20 RA patients, reported reduced inflammation (measured by lower GI scores, %BOP, and serum levels of MMP-3, IL-6, and TNF-α inflammatory markers) as well as reduced PPD [53]. Similar positive findings were reported for tocilizumab, an IL-6 receptor-blocking monoclonal antibody also approved for RA. A controlled clinical trial found significant improvements in GI scores, %BOP, PPD, clinical attachment level (CAL), and reductions in circulating serum levels of MMP-3 and IL-6 [52]. In a separate in vivo study, tocilizumab reduced alveolar bone loss and was associated with time-dependent decreases in the expression of pro-inflammatory IL-6, IL-17, and RANKL genes [21].

Evidence for rituximab, a monoclonal antibody used to treat RA and certain lymphomas by targeting the B-lymphocyte CD20 surface antigen, was more limited. A single study reported significantly reduced PPD and AL in two patients diagnosed with moderate periodontitis at baseline, both of whom showed no signs of active periodontitis at follow-up [49]. The remaining animal studies investigated specific mechanisms related to periodontal health using different inhibitors. Fluoxetine, a selective serotonin reuptake inhibitor with known anti-inflammatory properties [48]; odanacatib, a cathepsin K inhibitor investigated for osteoporosis [51]; and 1-trifluoromethoxyphenyl-3-(1-propionylpiperidin-4-yl) urea (TPPU), a soluble epoxide hydrolase inhibitor [20], each demonstrated reduced bone loss and favourably modulated the immune response [20, 48, 51]. In contrast, etanercept, a TNF inhibitor which is also used to treat RA, did not improve or worsen any reported outcomes [50].

Two observational studies [56, 58], nine randomised controlled trials (RCTs) [22, 28, 32, 33, 57, 59–62], and one non-RCT [58] evaluated the effects of doxycycline at sub-antimicrobial doses (20 mg twice daily [22, 28, 32, 33, 56, 57, 59–62], and 20 mg or 30 mg twice daily [58]). The most frequently reported outcome measures were PPD (in all studies), AL [22, 28, 32, 33, 56, 57, 61, 62], and %BOP [22, 28, 32, 57, 60–62]. Administration of doxycycline at sub-antimicrobial dose was associated with reductions in PPD and AL, as well as decreased %BOP. Additional outcomes included alterations in gingival microflora and collagenase activity, suggesting a beneficial effect on the biological mechanisms underlying PD. Overall, these trials support the efficacy of doxycycline at sub-antimicrobial dose in reducing key clinical indicators of PD severity. In a follow-up observational study, Caton et al. (2001) showed PPD reductions 3 months post-treatment, suggesting sustained therapeutic benefits [56]. However, in two shorter-duration trials by Golub et al. (1990), conducted over two weeks in a small number of participants, reductions in gingival crevicular fluid collagenase activity were reported, but without significant improvements in PPD [58].

Regarding immunisation agents, O’Brien-Simpson et al. (2016) explored six immunisation agents in mice; all significantly reduced mean alveolar bone loss compared to the control group. Among them, the peptide-based immunogen, KAS2-A1, consistently reduced alveolar bone loss through a suggested Th2-mediated immune response – see Supplementary file 2. In Experiment 4, it conferred prophylactic protection against bone loss induced by *P.g.* alone and in combination with *T.d.* and *T.f*. Experiments 5 and 6 demonstrated that both passive prophylactic and therapeutic immunisation with KAS2-A1 antibodies out-performed non-specific antibodies, supporting its potential as a candidate for effective PD prevention and treatment. Separately, Sugano [63] conducted a RCT in adults with untreated periodontitis using anti-*P.g.* egg yolk antibody against gingipains (IgY-GP). In this RCT, patients who received IgY-GP exhibited a significantly greater reduction in PPD compared to controls, but no significant difference was observed in %BOP, suggesting an improvement in connective tissue attachment with no measurable change in gingival inflammation.

Only four immunomodulatory agents were evaluated as adjuncts to conventional periodontal therapy: IgY-GP, assessed in one RCT [63]; PGRN-GelMA complex [12] and CCL22 [23], each evaluated in vivo in a dog model; and doxycycline at a sub-antimicrobial dose. Notably, doxycycline was the only agent studied both as an adjunct (*n* = 9 RCTs, *n* = 1 clinical trial and *n* = 1 observational study [22, 28, 32, 33, 56–62]) and as a standalone treatment (*n* = 1 uncontrolled clinical trial [58]). No other immunomodulatory agents were evaluated against standard periodontal treatment.

## Discussion

Immunomodulatory agents for the treatment of PD have been investigated using a wide range of methodologies, including observational studies, clinical trials, in vivo animal models, and in vitro cell lines. Pre-clinical studies have explored the underlying biological mechanisms of PD. Among the human studies, several have evaluated agents originally approved for the treatment of RA, an inflammatory joint condition with recognised association with PD [2, 4, 6]. Several other clinical studies have investigated doxycycline, a broad-spectrum antibiotic with decades of clinical use in the general management of bacterial infections [61, 70]. Together, these varied study designs have advanced our understanding of PD pathogenesis and potential therapeutic strategies relevant to clinical care.

### Tetracyclines at sub-antimicrobial doses

Sub-antimicrobial dose doxycycline (SDD) emerged as the most clinically researched immunomodulatory agent. At sub-antimicrobial doses, doxycycline inhibits endogenous collagenases, including MMPs, thereby modulating connective tissue breakdown without promoting antimicrobial resistance [28, 32, 33, 57–60]. One study also proposed an alternative mechanism involving inhibition of reactive nitrogen species [61].

SDD has been evaluated across a range of patients with periodontitis of varying severity, including adults of different ages and geographic locations [22, 32, 33, 58, 59, 61]. Specific high-risk groups studied included post-menopausal women [62], adult smokers [60], adults undergoing full-thickness flap surgery to treat PD [58], and adults with co-morbid PD and diabetes mellitus [57]. These populations represent clinically relevant targets, as diabetes mellitus, tobacco smoking, and oestrogen deficiency are established risk factors for poorer periodontal outcomes [23, 50, 52, 60, 71].

In terms of clinical efficacy, periodontitis outcomes were mixed. Improvements in PPD, an indicator of active inflammation, were reported in the majority of studies [22, 28, 32, 33, 56, 57, 61], while few reported no significant difference [58–60, 62]. Gains in AL, a measure of historical and current hard tissue destruction, were more variable, with four studies reporting significant improvements [22, 28, 32, 59], and five studies showing no significant change [33, 56, 57, 60, 62]. Only two RCTs [22, 32] administered a longer 9-month SDD therapy and met thresholds for clinical relevance, including changes in PPD or CAL by ≥2 mm or ≥3 mm within one year, or sites with probing depth ≤ 3–5 mm after therapy [32, 72]. These findings suggest that regular and prolonged therapy may be required for clinically detectable outcomes. This is supported by findings from a meta-analysis which reported significantly improved PPD and AL in smokers following 9 months of adjunctive SDD therapy [73], contrasting with an individual study that reported no benefit following 3-month adjunctive SDD in smokers [60].

Gingival inflammation outcomes generally showed limited response to adjunctive SDD therapy. The most assessed outcome measure was %BOP, with the majority of RCTs reporting no significant change [28, 57, 60, 62]; only two RCTs reported a significant reduction [32, 61]. Findings for the MGI score were similarly mixed: one RCT [33] and one non-controlled trial [58] reported significant reductions in inflammation, while another found no significant change [59].

Based on the available evidence, SDD may be a useful adjunct for the treatment of chronic PD, including in higher-risk patients such as those receiving surgical management for periodontitis and patients with diabetes mellitus [57, 58, 71]. However, consideration should be given to safety and bias as, while generally well-tolerated, adverse effects have been reported. A serious adverse event (dizziness), considered to be doxycycline-related, was reported in one study [32], and a RCT recorded participant withdrawals due to headache, indigestion, and rash [59]. Several authors also disclosed conflicts of interest, including affiliations with pharmaceutical patents, notably CollaGenex Pharmaceuticals, who manufacture Periostat^®^, the SDD formulation used in some RCTs [22, 32, 33, 59, 60, 62].

### Selective pharmacological inhibitors

Several of the reported selective pharmacological inhibitors are already used in clinical practice, most notably for RA, but also for diabetes and depression. Of these, only four agents – infliximab, rituximab, adalimumab, and tocilizumab – were studied in human populations, with the remainder assessed in pre-clinical animal models [38, 49, 52–55]. These four RA-targeted medications act via distinct inflammatory pathways that are also implicated in PD: infliximab and adalimumab inhibit the pro-inflammatory cytokine TNF-α [38, 53–55]; rituximab depletes B cells by targeting CD20, a surface protein expressed during intermediatory B cell development, thereby preserving B cell synthesis and long-term humoral immunity [74, 75]; and tocilizumab blocks the IL-6 receptor, interrupting downstream pro-inflammatory signalling [52].

Given the overlap in inflammatory mechanisms between RA and PD – including cytokine activity, immune dysregulation, and bone resorption – it is plausible that these RA medications can be of therapeutic benefit in periodontal management [38, 49, 52, 54, 55]. Findings from several studies support this hypothesis: tocilizumab, adalimumab, and rituximab were each associated with reduced periodontal inflammation or tissue destruction [49, 52, 53], and infliximab demonstrated similar effects in two of three studies [54, 55]. These findings reached a clinically relevant threshold for tocilizumab [52] and infliximab [55]. Furthermore, the mechanistic findings – reduced serum IL-6 and TNF-α with adalimumab [52], decreased TNF-α in gingival crevicular fluid with infliximab [54, 55], and downregulated IL-6, IL-17, and RANKL expression with tocilizumab [21] – are consistent with established disease pathways in both RA and PD, providing further support for their potential application in PD. Additionally, evidence from a prospective trial supports prolonged therapy duration, with 20-month therapy associated with significantly improved PD outcomes and lower serum levels of pro-inflammatory MMP-3 and IL-6, without adverse events, compared to 8-week therapy [52].

The generalisability of the findings is limited by small sample sizes (with the exception of the powered tocilizumab trial), presence of confounders [49], and lack of participant randomisation [54, 55]. Furthermore, TNF-α inhibitors are generally discouraged for periodontal therapy due to the severe side-effect profile associated with their current infusion methods [55]. Nevertheless, these findings support the potential use of selective inhibitors in patients with periodontitis and co-morbid inflammatory conditions. Future research should investigate alternative dosing strategies or related agents with similar selectivity but improved safety and administration profiles.

### Combination treatment

One in vivo exploratory study, also funded by CollaGenex Pharmaceuticals, investigated a combination immunomodulatory approach using a rat model of infection-induced periodontitis [24]. Combined treatment with CMT-8 and a sub-optimal dose of clodronate, a bisphosphonate drug, was associated with significantly less alveolar bone loss compared to CMT-8 or clodronate alone; notably, the bone levels measured were comparable to those of the healthy control group [24]. However, a separate study investigating bisphosphonates for the treatment of PD found no significant benefit in preventing alveolar bone resorption [51]. Furthermore, clinical use of bisphosphonates, particularly in the dental setting, is controversial due to the increased risk of medication-related osteonecrosis of the jaw [51]. Overall, there is a potential for combination therapy to improve PD treatment, however, extensive research is required to identify synergistic agents and evaluate their safety.

### Immunisation agents

Vaccinations may be a particularly useful approach if they can target the keystone pathogens that induce gingivitis and periodontitis [17]. Two articles investigated vaccination strategies for the management of PD; one in a RCT of patients with periodontitis [63], and the other in a series of mouse models of infection-induced periodontitis [17]. In both studies, the researchers employed immunisation strategies targeting the gingipains of *P. gingivalis*, a key virulence factor recognised to contribute to the pathogenicity of this bacterium [15, 17, 19, 39].

In the RCT by Sugano [63], the adjunctive IgY-GP oral vaccine reportedly reduced the number and level of *P.g.* with an associated significant reduction in PPD, although not reaching a clinically relevant threshold. This RCT was only reported as an abstract submission, and the full article could not be located. Meanwhile, in the mouse models conducted by O’Brien-Simpson et al. [17], six promising immunisation agents were identified, and the lead candidate, the KAS2-A1 immunogen, significantly reduced alveolar bone loss in both prophylactic and therapeutic regimens [17]. While encouraging, these pre-clinical findings are limited by the use of single- and tri-pathogen models of periodontitis, relatively short follow-up duration (62 days), and potential conflicts of interest due to industry funding (Sanofi Pasteur, vaccine manufacturer).

Beyond this review, gingipain-targeted vaccines have shown efficacy in significantly reducing alveolar bone loss in a primate model, although this study was limited by a small sample size [76]. Additional pre-clinical mouse studies have reported beneficial effects with other gingipain-related immunogens, such as RgbA-Kgb complex [77], RgbA, and heat-killed *P.g.* [78]. Since the RgpA-Kgp complex is significantly positively correlated with the severity of PD, targeting this gingipain may be particularly effective for individuals with aggressive periodontitis [79]. Nonetheless, vaccine development faces several challenges, including the presence of multiple *P.g.* strains, diversity in bacteria surface antigens, and difficulties in administration routes, including effective oral delivery due to the risk of protein degradation [80].

### Other agents tested in vivo and in vitro

Many immunomodulatory agents reviewed have only been tested in vitro or in animal models, which limits conclusions about their clinical applicability. However, these studies provide valuable insights into underlying mechanisms and help identify promising candidates for future research.

#### Tricyclic antidepressants

Desipramine and tianeptine demonstrated positive PD outcomes in a ligature-induced animal model; however both caused weight loss after short-term oral administration (15 days for desipramine; 21 days for tianeptine) [41, 67]. In humans, oral tricyclic antidepressants are known to cause a wide range of sustained and sometimes life-threatening side-effects when used to treat major depression, resulting in low patient tolerance and adherence [81]. Local administration may reduce these side effects [82, 83], but this approach requires rigorous pre-clinical evaluation to confirm its safety and efficacy.

#### Anti-hyperglycaemic drugs

In this review, evidence regarding the use of anti-hyperglycaemic drugs for managing periodontitis was limited. Available studies suggest these agents may modulate pro-inflammatory pathways, particularly those involving the IL-1β cytokine [3, 64]. Stronger evidence exists in the broader literature for gliclazide and metformin. In a rat model of ligature-induced periodontitis, gliclazide reportedly attenuated alveolar bone loss at doses of 1 and 5 mg/kg but exacerbated it at 10 mg/kg, indicating a potential toxicity threshold [84]. Metformin has been more extensively studied: a retrospective cohort study reported a significant, dose-dependent reduction in the risk of gingivitis and periodontitis among diabetic patients taking metformin [85]. Additionally, two meta-analyses demonstrated that adjunctive use of metformin gel with PMPR significantly improved clinical outcomes, including probing depth reduction (by 2.12–3.11 mm) and clinical attachment gain (by 2.29–2.83 mm), reaching thresholds considered clinically relevant [82, 83]. Therefore, gliclazide and metformin are promising immunomodulatory agents for PD, particularly in diabetic patients who may be prescribed these drugs for the treatment of hyperglycaemia, a common diabetic complication [3, 64, 82, 84]. However, the optimal non-toxic dosing of gliclazide requires further study; for metformin, while one meta-analysis noted limited safety data [82], another reported no adverse effects across five RCTs evaluated [83]. Future studies should continue to confirm safety profiles and assess efficacy in periodontal contexts.

#### Polymer-loaded nanofibres and micro-RNA based agents

Emerging evidence supports the potential of innovative delivery systems to improve the performance of immunomodulatory therapies for periodontitis. Poly(ɛ-caprolactone) delivered via nanofibre membranes reduced alveolar bone loss and promoted osteogenesis in vivo, with the most pronounced effects seen using nanofibres of highly oriented structure, likely due to macrophage polarisation towards their reparative M2 phenotype [11]. Similarly, exosomal delivery of miR-126 modulated macrophage phenotype and reduced inflammation and bone loss in vivo, particularly when exosomes were engineered to overexpress CXCR4 for targeted delivery [68]. Although these findings are pre-clinical, they align with wider trends in regenerative medicine and oncology where nanofibre scaffolds and exosome-based therapies are being explored to enhance tissue repair and drug delivery [86, 87]. These promising findings support further research into next-generation, low-toxicity delivery strategies in immunomodulatory periodontal therapy, however clinical translation remains to be established.

#### Plant-derived agents

Three plant-derived compounds – 6-shogaol (from dried ginger) [2], CsinCPI-2 (a cysteine peptidase inhibitor from Citrus sinensis) [7], and boldine (from the boldo tree) [31] – were reported to prevent alveolar bone loss in vivo. Regarding the potential underlying mechanisms, CsinCPI-2 and boldine reduced osteoclast numbers in vivo. 6-shogaol had the same effect on osteoclasts in vitro. Additionally, when investigated in vivo, 6-shogaol downregulated IL-1β and TNF-α levels, whereas boldine was associated with reduced RANKL and Th17 cell activity, alongside increased osteoprotegerin and Treg cell activity, indicating a broader anti-inflammatory effect (osteoprotegerin is a protein which binds to RANKL to suppress its osteoclastogenic activity) [88]. In a further in vivo study, 6-shogaol tested at double the dose (20 mg/kg/day) also prevented alveolar bone loss [89]. No adverse events were reported for 6-shogaol and CsinCPI-2 [7, 89].

Cannabinoids have regulatory approval in various regions for the treatment of anorexia, Parkinson’s disease and chemotherapy-induced nausea [90]. In the context of PD, cannabinoids showed modulation of multiple cytokines in vitro – see Supplementary file 2 [1]. In a ligature model of periodontitis, daily cannabidiol treatment reduced alveolar bone loss [91].

Overall, early research into plant-derived agents such as cannabinoids, 6-shogaol, and boldine demonstrates potential anti-inflammatory and bone-protective effects relevant to PD. While cannabinoids already have established clinical use and safety profiles, 6-shogaol and boldine show encouraging pre-clinical results to support further investigation.

#### Vitamin-based agents

Three vitamin-based agents have shown immunomodulatory effects relevant to PD. ATRA, an active metabolite of vitamin A, demonstrated anti-inflammatory and bone-protective effects in vivo [16]. Similarly, the synthetic derivative of ATRA, fenretinide, demonstrated anti-inflammatory effects in vitro [66]. Both fenretinide and ATRA have shown promise in anti-cancer therapies [92, 93], and fenretinide is currently being investigated for the treatment of neurological diseases due to its low toxicity and ability to modulate several biological pathways [92].

L-ascorbic acid 2-phosphate magnesium salt (APM), derived from vitamin C, demonstrated greater anti-inflammatory effects in vitro than standard vitamin C through mechanisms involving type I collagen synthesis and TNF-α activity (see Supplementary file 2) [65]. Clinical and pre-clinical evidence supports its potential effectiveness and safety: a RCT reported significantly reduced gingival inflammation with an APM-containing dentifrice without adverse events [94], while local sub-periosteal administration of vitamin C significantly protected against alveolar bone loss and AL in diabetic rats with periodontitis [95].

#### Chemokine-based agents

CCL22, a known chemoattractant for the anti-inflammatory Treg cell, was evaluated in three animal models, all of which reported reduced alveolar bone loss [23]. Non-toxicity was observed at all tested doses (25 mg/ml orally in mice; 2-4 mg into periodontal pockets in beagle dogs). Immunomodulatory effects included downregulated expression of RANKL and pro-inflammatory cytokines (IL-1β, IFN- γ, TNF-α and IL-17), alongside upregulated expression of bone pro-regenerative factors and Treg-specific anti-inflammatory proteins (Foxp3, IL-10, TGF-β and CTLA-4). While these findings are encouraging, further research is indicated as it was unclear if treatment allocation was randomised or blinded, and the sampling power was not reported.

#### Beta-glucans

Beta-glucans are naturally occurring polysaccharides found in yeast, oats, and mushrooms, commonly consumed as dietary supplements and believed to possess immunomodulatory properties relevant to wound healing, infection, and cancer [96]. Beta-1,3/1,6-glucan, a carbohydrate polymer isolated from the cell wall of *Saccharomyces cerevisiae*, prevented alveolar bone loss with associated upregulation of corticosterone and TGF-1β in vivo [69]; TGF-1β, a cytokine synthesised by Treg cells and M2 macrophages, has an anti-inflammatory role in PD which includes downregulation of MMPs and RANKL [11, 12, 16, 23, 31]. This study employed a rat model and demonstrated robust methodology, including examiner blinding, randomised allocation, and a large sample size, although it is unclear if the study sample was powered. Other animal studies have reported beta-1,3/1,6-glucan, at higher doses (20-80 mg/kg vs. 10 mg/kg), prevented alveolar bone loss in the diabetic population, with no dose-dependent effect [97, 98]. The safety profile of beta-1,3/1,6-glucan appears favourable with no adverse effects reported [69, 99], although a lack of longitudinal data is recognised as a current limitation [100].

#### Strontium ranelate

This anti-osteoporotic drug significantly reduced alveolar bone loss at 900 mg/kg in vivo, a dose higher than that recommended for humans (2 g/day in humans, equivalent to 625 mg/kg in rats) [8, 101]. Furthermore, known systemic side effects (nausea, diarrhoea and arrythmia) would contraindicate its clinical suitability for periodontal therapy [102].

#### Growth factors

PGRN, when administered as a complex with GelMA to prolong its activity, significantly improved PD outcomes in vivo – specifically alveolar bone quantity, periodontitis markers (PPD and AL) and gingivitis markers (bleeding index) – with associated reductions in M1 macrophage & Th17 cell-associated markers, and elevated M2 macrophage & Treg cell-associated markers, indicating potential mechanisms of action. Despite these positive outcomes, the authors reported evidence that GelMA may promote inflammation, potentially leading to underestimation of PGRN effectiveness. Another in vivo study similarly reported reduced alveolar bone loss following treatment with recombinant human PGRN alone [103]. These findings support the anti-inflammatory activity of PGRN, consistent with reports for other conditions such as RA and inflammatory bowel disease [104], and highlight its potential as a therapeutic candidate for the treatment of PD. Further research is needed to confirm these effects.

### Limitations

Safety assessments are of particular concern for immunomodulatory agents as altering the immune response may have unintended consequences. In the context of PD, the safety profile for several agents – glyburide, gliclazide, fluoxetine, rituximab, etanercept, TPPU, boldine, cannabinoids, ATRA, fenretinide, CCL22, poly(ɛ-caprolactone) and the immunisation agents – remain unclear. Notably, the selective pharmacological inhibitor odanacatib was discontinued due to an increased risk of stroke [105]. Concerning side-effects have also been documented for TNF-α inhibitors, strontium ranelate, and oral tricyclic antidepressants. Local administration methods may help minimise side effects and warrant further exploration [82, 83]. Overall, while several studies show promising results, there remains a need for additional well-designed research, including robust clinical trials and comprehensive safety evaluations.

As more than half of the included studies were pre-clinical, the clinical relevance of their findings are limited. While cell cultures offer valuable mechanistic insights, they cannot replicate complex biological features of PD. Although animal models provided more substantial evidence, several models induced periodontitis by injecting a single or limited number of periodontal pathogens which does not reflect the multi-pathogenic nature of PD. The ligature-induced periodontitis models better mimic the natural pathophysiology, but the lack of measurements relating to clinical periodontal parameters made it challenging to assess the direct relevance of the tested agents in this context. Some studies also lacked methodological details such as sample size calculations, randomisation, blinding and dosing regimens, which may affect the reliability and external validity of their findings (see Supplementary file 2 for details). Only a few agents – doxycycline, IgY-GP, CCL22 and PGRN-GelMA – were directly compared to conventional periodontal therapy. As most other agents have not been evaluated against standard treatment, their clinical application remains uncertain. Furthermore, many RCTs, particularly those investigating doxycycline, were funded by the same company, introducing potential reporting bias; however not all authors disclosed funding sources or conflicts of interest.

This review included only English-language publications from high-impact journals accessible through institutional resources. Relevant studies published in other languages may be under-represented, introducing potential selection bias. This review was not pre-registered; a retrospective protocol documenting the full methodology is available in the Research Registry [106]. The interchangeable use of “immunosuppressive” and “immunomodulatory”, despite their distinct definitions, necessitated careful judgement during screening and may have affected study selection. Additionally, incomplete reporting across the studies limited the assessment of potential bias domains, including selection, performance, detection and reporting bias. The substantial heterogeneity of the evidence base – including study design, populations, interventions, outcome measures and follow-up durations – precluded quantitative synthesis and limits comparability. Although gingivitis was frequently assessed, variation in scoring systems and the absence of standardised clinical thresholds made it difficult to determine any meaningful effect on gingival inflammation.

## Conclusions

Evidence supporting immunomodulatory therapies for the management of periodontal disease remains limited, with few agents evaluated clinically. Only a small number of agents, including metformin, tocilizumab and infliximab, have demonstrated improvements in patients with co-morbidities, such as diabetes or rheumatoid arthritis, suggesting a potential role for multi-disciplinary care. For most agents, there is insufficient evidence of benefit beyond conventional periodontal treatment, and safety concerns further limit clinical applicability. Future research should prioritise well-powered, methodologically robust trials with appropriate comparators and standardised outcomes, while investigating whether prolonged therapy, alternative delivery strategies or combination approaches can offer meaningful clinical benefit.

## Supplementary Information

Supplementary File 1.

Supplementary File 2.

## Data Availability

All data analysed during this study are included in this published article and its supplementary information files.

## Abbreviations

APM: L-ascorbic acid 2-phosphate magnesium salt
AL: Attachment loss
ATRA: All-trans retinoic acid
%BOP: Percentage sites with bleeding on probing
CAL: Clinical attachment level
CCL22: Recombinant mouse C-C motif chemokine ligand 22
CMT-8: 6a-deoxy,5 hydroxy-4-dedimethylaminotetracycline (abbreviated as chemically modified non-antimicrobial tetracycline)
CXCR4: C-X-C chemokine receptor type 4
CXCR4-miR126-Exo: CXCR4-overexpressing exosome loaded with miR-126
GelMA: Gelatin methacryloyl
GI: Gingival index
IgY-GP: Anti-Porphyromonas gingivalis egg yolk antibody against gingipains
IL: Interleukin
MGI: Modified gingival index
MMP: Matrix metalloproteinase
PD: Periodontal disease
P.g.: Porphyromonas gingivalis
PGRN: Progranulin
PLN: Periodic lamellae structure
PMPR: Professional mechanical plaque removal
PPD: Probing pocket depth
RA: Rheumatoid arthritis
RANKL: Receptor activator of nuclear factor kappa-B ligand
RCT: Randomised controlled trial
SDD: Sub-antimicrobial dose doxycycline
SRP: Scaling and root planing
T.d.: Treponema denticola
T.f.: Tannerella forsythia
TPPU: 1-trifluoromethoxyphenyl-3-(1-propionylpiperidin-4-yl) urea
