# Supplementary material for: Immunomodulation as a novel treatment strategy for periodontal disease: a systematic review
Source: BMC Oral Health. 2026 Jun 15;26:1724. doi: 10.1186/s12903-026-08120-0 (PMC13573343; doi:10.1186/s12903-026-08120-0)
Supplement: Supplementary file 2 [file 12903_2026_8120_MOESM2_ESM.docx]

| Table S1. Characteristics and key findings of studies included in the systematic review | | | | | |
| --- | --- | --- | --- | --- | --- |
| First author (year) | **Intervention** | **Control** | **Study design and protocol** | **Outcomes measured** | **Significant findings^a^** |
| *Tetracyclines at sub-antimicrobial doses* | | | | | |
| Caton *et al.* (2000);  Study 1 | SDD Group (n=94): sub-antimicrobial dose doxycycline (SDD), 20mg. Twice daily oral administration (once in morning & once in evening, 12 hours apart, 1 hour before eating) for 9 months. | Placebo Group (n=96): identical placebo. Twice daily oral administration (same instruction as intervention). | Randomised controlled trial.  Population: 30-75-year-old adult patients with periodontitis, USA, N=190 (NPC: sample size calculation or power analysis not reported).  Duration: 9 months.  Protocol: double-blinded. All patients received scaling and root planing (SRP) prior to trial.  Measurements at month 3, 6 and 9. | Probing pocket depth (PPD); Attachment loss (AL); percentage (%) sites with AL and PPD gain ≥2mm or ≥3mm; % bleeding on probing (%BOP); change in periodontal microflora (including spirochetes, periodontal pathogens and doxycycline-resistant bacteria) in sub-gingival plaque | Lost to follow-up: n=19 patients (n=7 SDD Group, n=12 Placebo Group). Withdrawals due to adverse events: n=1 SDD Group, n=2 Placebo Group.  For baseline PPD 0-3mm: reduced PPD and lower %BOP at all time points.  Baseline PPD 4-6mm: reduced AL and PPD at all time points; lower %BOP; time-dependent.  Baseline PPD ≥7mm: reduced AL and PPD at all time points; reduced % sites with AL ≥2mm at month 9.  Reduced small spirochetes at months 3 and 6, intermediate spirochetes at month 9, large spirochetes at month 6. No difference for periodontal pathogens. |
| Caton *et al.* (2002);  Study 2: Follow-up study to Caton *et al.* (2000) | SDD Group (n=77): no additional treatment allocated. | Placebo Group (n=74): no additional treatment allocated. | Observational study.  Population: adult patients with periodontitis, USA, N=151 (NPC).  Protocol: double-blinded.  Measurements at month 9 and 12. | AL; PPD; periodontal microflora in sub-gingival plaque | Lost to follow-up: n=5 SDD Group in original study.  For baseline PPD 4-6mm and ≥7mm: reduced PPD sustained at month 12. |
| Choi *et al.* (2004) | SDD Group (n=15): SDD, 20mg oral tablet, twice daily. | Placebo Group (n=17): placebo oral tablet, twice daily. | Randomised controlled trial.  Population: 25-to-64-year-old adult patients with chronic periodontitis, South Korea, N=32 (n=17 male; NPC).  Duration: 120 days.  Protocol: double-blinded. All received SRP prior to trial. | PPD; AL; BOP; matrix metalloproteinase (MMP)-8 levels in gingival crevicular fluid (GCF);  gingival expression of MMP-9, tissue inhibitor of metalloproteinase (TIMP)-1 and interleukin (IL)-6 | Reduced PPD, AL and MMP-8 levels. Higher TIMP-1 expression. |
| Emingil *et al.* (2004) | Low-dose doxycycline (LDD) Group (n=10, n=5 male): LDD, 20mg capsule, twice daily for 3 months, (instructed to take one in morning & one in evening, 1 hour before meals). | Placebo Group (n=10, n=6 male), placebo capsule, twice daily for 3 months (same instruction as intervention). | Randomised controlled trial.  Population: 37-to-61-year-old adult patients with chronic periodontitis, Turkey, N=30 (n=20 male; NPC).  Protocol: double-blinded. All patients received SRP and oral hygiene instruction (OHI) as required at baseline. OHI reinforced and maintenance therapy performed at month 3, 6, 9 and 12. | PPD; AL; gingival index (GI) score; plaque index (PI) score; GCF levels of MMP-8 | Withdrawals: n=10 (unable to attend appointments).  Reduced PPD and GI score at all time points, and reduced MMP-8 levels at month 6 only. |
| Gilowski *et al.* (2012) | SDD Group (n=17): SDD, 20mg, twice daily administration, (instructed to take one morning and one in evening, 1 hour before meals). | Placebo Group (n=17): indistinguishable placebo, twice daily (same instruction as intervention). | Randomised controlled trial.  Population: 36-to-68-year-old adult patients with moderate-to-severe chronic periodontitis and optimally-controlled diabetes mellitus, Poland, N=34 (n=16 male; PC: power calculation reported).  Duration: 3 months.  Protocol:  double-blinded. Non-blinded statisticians. All patients received full-mouth SRP and OHI prior to trial. | PPD; AL; %BOP; approximal PI; GCF volume; MMP-8 concentration and amount in GCF | For baseline PPD ≥4mm: reduced PPD.  Non-significant findings of note: AL and %BOP reduced in both groups, while approximal PI, GCF volume and total MMP-8 reduced in SDD Group only. |
| Golub *et al.* (1990);  Study 1 | LDD Group (n=4): LDD, twice daily, 20mg capsule, during first 24 hours (loading dose), followed by 30mg capsule (maintenance dose); instructed to take one morning and one evening, avoiding mealtimes. | Placebo Group (n=4): placebo oral capsule, twice daily. | Clinical trial.  Population: 35-to-56-year-old adult patients with moderately severe chronic periodontitis requiring full-thickness flap surgery, USA, N=8 (NPC).  Duration = 2 weeks.  Protocol: non-randomised, single-blinded allocation. Blinded analyser. All participants received standardised OHI and SRP for maxillary right and left posterior sextants prior to surgery. | Collagenase activity in maxillary gingivae; GI score; PPD | Reduced gingival collagenase activity. |
| Golub *et al.* (1990);  Study 2 | LDD N=4: 20mg capsule, twice daily, (instructed to take one morning and one evening, avoiding mealtimes). | None. | Clinical trial.  Population: 35-to-56-year-old adults with moderately severe periodontitis, USA, N=4 (NPC).  Duration = 2 weeks.  Protocol: non-blinded. | Collagenase activity in GCF; GCF flow; GI score; PPD | Reduced GCF flow at weeks 1 and 2. Reduced GCF collagenase activity and GI score at week 2 only. |
| Golub *et al.* (2001) | SDD (doxycycline hyclate, specially formulated), 20mg oral capsule (n unreported):  Part I:   - Group 1: twice daily weeks 0-12; - Group 2: once daily weeks 0-12; - Group 3: twice daily weeks 0-4, once daily weeks 5-12; - Group 4: twice daily weeks 0-4.   Part II: no treatment (all Groups).  Part III: once daily, weeks 25-36 (Groups 1, 2 and 3). | Placebo: visually indistinguishable oral capsules (daily schedule, and group n unreported):  Part I:   - Group 4*:* weeks 5-12; - Group 5: weeks 0-12.   Part II: no treatment (all Groups).  Part III: weeks 25-36 (Groups 4 and 5). | Randomised controlled trial.  Population: 18-to-75-year-old adult patients with periodontitis, N=75 (NPC).  Duration: Part I: weeks 0-12; Part II: weeks 13-24 (no treatment; scaling & prophylaxis repeated); Part III: weeks 25-36 (intervention or control once daily).  Protocol: double-blinded. Prior to trial, patients received supra- and sub-gingival scaling & prophylaxis and OHI. | Collagenase activity in GCF; AL; PPD; GCF flow; severity of whole-mouth gingival inflammation (quantified using modified GI and Eastman Interdental Bleeding Index) | Withdrawals: n=9 during Part I (3 possible SDD-related adverse events, 1 disqualified, 5 unrelated), n=15 during Parts II and III for administrative reasons.  Part I: Reduced collagenase activity in Group 1 compared to Group 5. Group 4 changed from SDD to placebo, showed collagenase activity increased to a level comparable to Group 5 at week 12.  Part II and III: Reduced AL in Group 1 compared to Groups 2-5. |
| Needleman *et al.* (2007) | LDD Group (n=17): LDD, 20mg oral (Periostat®), twice daily for 3 months. | Placebo Group (n=18): identical oral placebo, twice daily for 3 months. | Randomised controlled trial.  Population: 32-to-58-year-old adult smokers with chronic periodontitis, UK, N=35 (PC).  Duration: 6 months  Protocol: triple-blinded. Blinded analyser. All received SRP & OHI prior to trial; supportive periodontal therapy provided at recalls.  Measurements at months 1, 3 and 6. | Primary: AL. Secondary: PPD; gingival recession;  %BOP; carboxyterminal telopeptide of type I collagen (ICTP) levels in GCF.  Explanatory: plaque score (%) | Withdrawals; n=1 prior to trial (LDD), n=4 during trial (n=2 LDD, n=2 placebo) for reasons unrelated to intervention.  All between-group differences were non-significant. |
| Parvu *et al.* (2013) | SDD Group (n=87): SDD, 20mg oral capsule, twice daily. | Placebo Group (n=87): cornstarch capsule, twice daily. | Randomised controlled trial.  Population: adults with untreated moderate-to-advanced chronic periodontitis, Romania, N=174 (NPC).  Duration: 3 months.  Protocol: double-blinded. All received SRP prior to treatment, and weekly root planing for first 4 weeks of trial. Thereafter, no SRP. | Oedema, fibrosis, necrosis and dysplasia in gingival tissue; inflammatory cell influx in gingival tissue; expression of inducible nitric oxide synthase (iNOS) and 3-nitrotyrosine (3NT); serum levels of nitrite (NO_2_^-^), nitrate (NO_3_^-^) and protein-bound 3NT adduct; PPD; %BOP; AL | Withdrawals: none.  Reduced PPD, %BOP and 3NT expression. Fewer iNOS-positive leukocytes. Significant correlation between iNOS expression and clinical indices in SDD Group only. |
| Preshaw *et al.* (2004) | SDD Group (n=107): SDD, 20mg tablet, twice daily (instructed to take one morning and evening, 1 hour before meals). | Placebo Group (n=103): visually identical tablet, twice daily (same instruction as intervention). | Randomised controlled trial.  Population: adult patients with moderate-to-severe chronic periodontitis, USA, N=210 (PC).  Duration: 9 months.  Protocol: double-blinded. All received SRP prior to treatment. Periodontal pockets with ≥2mm attachment loss received additional periodontal therapy.  Measurements at months 3, 6 and 9. | AL; % sites with clinical attachment gain ≥2 and ≥3mm; PPD; % sites with PPD reduction ≥2 and ≥3mm (PPD: 1-3mm = “normal”, 4-6mm = “mild-to-moderate”, ≥7mm = “severe”) | Withdrawals: n=1 in Placebo Group before trial, n=52 during trial (Adverse event; Illness not related to drug; Protocol deviation; Lost to follow-up; Other; n reported in Table 2 of study).  Baseline PPD 4-6mm: reduced AL at month 9 and reduced PPD at months 3, 6 and 9. Baseline PPD≥7mm: reduced AL and PPD at months 6 and 9. Baseline PPD ≥4mm and ≥6mm: higher % sites with clinical attachment gain and PPD reduction of ≥2mm and ≥3mm. |
| Reinhardt *et al.* (2007) | SDD Group (n=64): SDD, 20mg tablet, twice daily. | Placebo Group (n=64): visually identical tablet, twice daily. | Randomised controlled trial.  Population: post-menopausal women aged 45-70 years with generalised moderate-to-advanced periodontitis, USA, N=128 (PC).  Duration: 2 years.  Protocol: triple-blinded. All received calcium & vitamin D daily supplements and periodontal maintenance therapy every 3-4 months. | PPD; AL; BOP; Supra-gingival plaque levels | Withdrawals: n=13 SDD Group, n=2 Placebo Group.  No significant differences for outcome measures.  Reported findings limited to post-hoc and selective analyses (e.g. exploratory regressions). |
| Selective pharmacological inhibitors | | | | | |
| Branco-de-Almeida *et al.* (2012) | Fluoxetine (a selective serotonin reuptake inhibitor [SSRI]) (n=10): with periodontitis, daily oral gavage for 15 days, 20mg/kg, in saline. | Two groups:  Healthy Controls (n=10): without periodontitis, no treatment.  Placebo Group (n=10): with periodontitis, daily oral gavage with saline for 15 days. | *In vivo* (Rat model).  Population: N=30 male Wistar rats (NPC).  Protocol: randomised allocation, blinding of allocation not reported. Blinded examiner. Ligature-induced periodontitis (left and right mandibular first molars).  Measurements at day 3 or 15 after ligature placement. | Alveolar bone loss; % area of collagen fibres in connective tissue; mRNA expression of IL-1β, cyclooxygenase (COX)-2, MMP-9 and iNOS after 3 days; MMP-9 activity after 3 days | Reduced alveolar bone loss compared to Placebo Group, greater bone loss compared to Healthy Controls. Greater collagen fibre content compared to Placebo Group. Elevated expression of IL-1β, MMP-9 and iNOS compared to Healthy controls. Reduced expression of IL-1β and COX-2 compared to Placebo Group. Elevated MMP-9 activity compared to Healthy Controls and reduced compared to Placebo Group. |
| Coat *et al.* (2015) | Rituximab (an anti-CD20 monoclonal antibody). Two groups:  Group II at baseline (n=10; 2 males), had previously received >2 courses of rituximab infusions.  Group I at follow-up (n=11; 4 males), treated with 2 x 1000mg infusions with concomitant 100mg methylprednisolone (to avoid immediate reactions as per clinical recommendations). | Group I at baseline (n=11; 4 males), no prior rituximab treatment. | Observational study.  Population: 47-to-83-year-old rheumatoid arthritis (RA) patients, France, N=21 (NPC).  Duration: main study 6 months, 48 month follow-up restricted to n=5 in Group I.  Protocol: blinded examiner.  Measurements at baseline for both Groups, and at follow-up for only Group 1. | Number of decayed, missing and filled teeth (DMFT); PI score; modified gingival index (MGI); Papillary bleeding index (PBI); PPD; AL; severity of periodontitis (American Academy of Periodontology (AAP) and Centers for Disease Control & Prevention classifications) | At baseline:   - Group I had lower PI score - Group I had higher MGI score, PPD and AL - Greater proportions in Group I had more severe periodontitis - No difference in DMFT or PBI score   At 6 months, for Group I:   - Reduced PPD and AL - No change in DMFT - No change in MGI, PBI or PI scores. Authors suggest observed improvements in PPD and AL are therefore not due to oral hygiene improvement - Periodontitis severity changed from moderate to no periodontitis for 2 patients. |
| Grauballe *et al.* (2015) | Etanercept (a tumour necrosis factor [TNF]-α inhibitor): 4.25mg/kg on average. Three times weekly subcutaneous injections, started 1 day after baseline, stopped end of week 5.  OPDE Group (n=15): obese, with periodontal disease (PD). | Saline subcutaneous injections (same schedule as intervention):   - Lean Control (LC) Group (n=18): lean, without PD; - Lean + PD (LPD) Group (n=17): lean, with PD; - Obese Control (OC) Group (n=15): obese, without PD; - Obese + PD (OPD) Group (n=15): obese, with PD. | *In vivo* (Rat model).  Population: obese diabetic male Zucker rats & 35 lean littermates, N=45 (NPC).  Protocol: randomised allocation, blinding of allocation not reported.  Ligature-induced PD (maxillary second molars).  Measurements at end of week 5. | Systemic inflammation (IL-1β, IL-6 and TNF-α levels in blood plasma); alveolar bone loss (ACJ-ABC distance); radiographic alveolar bone support (distance from any interproximal defect to the radiographic apex) | Lost to follow-up: n=11.  OPDE Group had greater alveolar bone loss and reduced alveolar bone support compared to OC Group. |
| Hao et al. (2015) | Odanacatib (a cathepsin-K inhibitor): weekly oral administration in dimethylsulfoxide (DMSO) for 63 days.  Three groups:  Group A (n=15): without periodontitis, 3.606mg/kg;  Group C (n=15): with periodontitis, 3.606mg/kg;  Group D (n=15): with periodontitis, 0.7212mg/kg. | DMSO: weekly oral administration.  Two groups:  Group B (n=15): without periodontitis;  Group E (n=15): with periodontitis. | *In vivo* (Mouse model).  Population: 7-to-8-week-old wild-type BALB/cJ mice, N=75 (NPC).  Protocol: randomisation/ (examiner) blinding protocol not reported. Infection-induced periodontitis (combination of *P.g.* W50, *T.d.* and *T.f.*).  Measurements at 56 days after initial infection. | Alveolar bone loss and periodontal ligament width; number of tartrate-resistant acid phosphatase (TRAP)-positive cells; % of toll-like receptor (TLR)4, TLR5- and TLR9-positive cells in gingival lesion areas; % of macrophages (F4/80-positive cells) and activated T cells (CD3-positive cells); mRNA expression levels of cathepsin K (CTSK), tumor necrosis factor (TNF)-α, IL-6, IL-23α, TLR4, TLR5 and TLR9; periodontal tissue protein levels of TNF-α, IL-6 and IL-23 | Group D reduced bone resorption compared to Group E, and Group C reduced bone resorption compared to Groups D and E (dose dependent).  Group A:   - Lower expression of TNF-α compared to Group E - Lower % of F4/80-, TLR4- and TLR9-positive cells than Group C   Group C:   - Reduced periodontal ligament width, fewer TRAP-positive cells, visually fewer osteoclasts, fewer % of F4/80-, CD3-, TLR4-, TLR5- & TLR9-positive cells, lower mRNA expression of CTSK, TNF-α, IL-23, IL-6, TLR-4, TLR-5 & TLR-9, and lower protein expression of TNF-α, IL-6 & IL-23 compared to Group E - Higher % of F4/80-positive cells than Group B |
| Kobayashi *et al.* (2014) | Tocilizumab (an IL-6 receptor inhibitor) (n=28).  Baseline: received tocilizumab for 20.3 ± 2.5 months before study start.  During study: 8 mg/kg tocilizumab, 4-weekly. | Control Group (n=27): no tocilizumab intervention prior to and during study. | Clinical trial.  Population: adult patients with RA and chronic periodontitis, Japan, N=55 (n=11 male; post hoc PC).  Duration: 8 weeks.  Protocol: non-randomised allocation. Blinded examiners. Periodontitis defined as at least one site with ≥4mm clinical attachment loss; severity per AAP classification.  Measurements at baseline and 8 weeks. | %BOP; GI score; PPD; % sites with PPD <4 and 4-6mm; AL; % sites with AL <4mm; number of teeth present; % number of sites with supra-gingival plaque; serum levels of: total immunoglobulin (Ig)G, IL-6, MMP-3, IgG antibodies against periodontopathic bacteria (*A.a.*, *E.c.*, *P.g.*, and *P.i.*) | At baseline, groups were comparable with respect to demographics, periodontal and rheumatological status.  At 8 weeks: reduced GI score, %BOP, PPD, % sites with PPD 4-6mm, AL, and serum MMP-3 & IL-6; higher % sites with PPD <4mm and with AL <4mm. |
| Kobayashi *et al.* (2014) | Adalimumab (a humanised anti-TNF-α monoclonal antibody): 40mg, fortnightly subcutaneous administration. | None. | Clinical trial.  Population: 28-to-67-year-old patients with RA, Japan, N=20 (n=1 male; PC).  Protocol: blinded examiners.  Measurements at baseline and 3 months. | %BOP; GI score; PPD; clinical attachment level; number of teeth present; % sites with supra-gingival plaque; serum levels of MMP-3, IL-6, TNF-α and various undisclosed proteins (to identify potential biomarkers) | Reduced GI score, %BOP, PPD, and serum levels of MMP-3, IL-6 and TNF-α. Plaque levels and clinical attachment level comparable to baseline. Reduced serum levels of potential biomarkers (complement factor H, phospholipase D, serum amyloid A, complement component 4, and α-1-acid glycoprotein) |
| Mayer, Balbir-Gurman and Machtei (2009) | RA+ Group (n=10): infusion of 200mg infliximab (an anti-TNF-α antibody) every 8 weeks. | Two groups:  RA- Group (n=10): with RA, no treatment;  Healthy Controls (n=10): without RA, no treatment. | Cross-sectional study.  Population: 22-to-76-year-old adults, Israel, N=30 (n=13 male; NPC).  Protocol: non-blinded clinical examiners, blinded laboratory testing.  Measurements at all teeth excluding third molars. | PPD; AL; PI score; GI score; %BOP; severity of PD using AAP classification; TNF-α levels in GCF | Reduced GI score and GCF TNF-α levels compared to RA- Group. Reduced PPD, %BOP and AL compared to RA- Group and Healthy Controls. |
| Mayer *et al.* (2013) | RA+ group (n=10): with RA, received infliximab, 3mg/kg infusion every 8 weeks (mean duration 26 ± 8 months). | Four groups, all no intervention:  RA Group (n=12): with RA;  PA Group (n=12): with psoriatic arthritis (PA);  SSc Group (n=12): with systemic sclerosis (SSc);  Healthy Controls (n=12): with PD, no autoimmune disease. | Cross-sectional study.  Population: 22-to-76-year-old adults, Israel, N=58 (NPC).  Protocol: non-blinded clinical examiners, blinded laboratory testing. | PPD; number of sites with PPD >4mm; PI score; GI score; Full-Mouth Bleeding Score (FMBS); PD severity (per AAP classification); TNF-α levels in GCF | Reduced PPD, number of sites with PPD >4mm, FMBS, GI score and GCF TNF-α levels compared to RA, PA and SSc Groups. Greater PI score than SSc Group and Healthy Controls. |
| Napimoga *et al.* (2018) | 1-trifluoromethoxyphenyl-3-(1-propionylpiperidin-4-yl) urea [TPPU] (a soluble epoxide hydrolase inhibitor). TPPU Group (n=6): with periodontitis, 1mg/kg dissolved in PEG400 (vehicle), daily oral gavage for 15 days. | Two groups:  Negative Control Group (n=6): without periodontitis, no treatment;  Positive Control Group (n=6): with periodontitis, daily oral gavage with PEG400 for 15 days. | *In vivo* (Mouse model).  Population: N=18 male mice (NPC).  Protocol: Randomisation/blinding of allocation not reported. Blinded examiner with randomised alveolar measurements. Infection-induced periodontitis (*A.a.*).  Measurements intervals reported including experiment end (15 days). | Alveolar bone loss (ACJ-ABC distance); expression of soluble epoxide hydrolase (sEH) and 84 genes involved in the host response to bacterial infection | Reduced alveolar bone loss and sEH expression compared to Positive Control Group. Six genes (*Il13, Il23, Apcs, Ifnβ, Mbl2* and *Mpo*) upregulated and 18 genes (*Cd8, Cd40Ig, Ccr6, Cd4, Ccr8, Lyz2, Tlr1, Stat4, IFNγ, Tlr9, Ccr4, Il5, Ifnα2, Cd40, Cxcr3, Gata3, Rag1* and *Mx1*) downregulated compared to Positive Control Group. |
| Pers *et al.* (2008);  Study 1 | Group I (n=20):  3mg/kg infliximab injection, every 6 weeks, taken with oral methotrexate (mean duration 22 months or longer). | Group II (n=20): no treatment. | Cross-sectional study.  Population: 31-to-76-year-old patients with RA, N=40 (n=9 male; NPC).  Protocol: blinded examiner. | DMFT score; presence of any removable or fixed prosthetic dental appliances; PI score; MGI score; PBI score; AL; PPD | Higher MGI and PBI scores, indicating Group I experienced more gingivitis. |
| Pers *et al.* (2008);  Study 2 | Infliximab, 3mg/kg injection, every 6 weeks, taken with oral methotrexate.  Group II from original study (N=9): RA patients. | None. | Follow-up clinical trial.  Population: N=9 patients from Study 1 (NPC).  Duration: 9 months.  Protocol: blinded examiner. | PI score; MGI score; PBI score; PPD; AL | MGI and PBI scores increased, indicating worsened gingival inflammation. AL decreased.  Non-significant findings of note: no difference in PPD or PI score, indicating improvement for AL was independent of oral hygiene. |
| Vieira *et al.* (2021) | Tocilizumab (TCZ): intra-peritoneal injection. Six groups, all with periodontitis:   - L-TCZ2-7 Group (n=9): 2mg/kg, sacrificed day 7; - L-TCZ2-14 Group (n=9): 2mg/kg, sacrificed day 14; - L-TCZ4-7 Group (n=9): 4mg/kg, sacrificed day 7; - L-TCZ4-14 Group (n=9): 4mg/kg, sacrificed day 14; - L-TCZ8-7 Group (n=9): 8mg/kg, sacrificed day 7; - L-TCZ8-14 Group (n=9): 8mg/kg, sacrificed day 14 | Without periodontitis:   - C-7 Group (n=9): sacrificed day 7; - C-14 Group (n=9): sacrificed day 14   With periodontitis   - L-7 Group (n=9): sacrificed day 7; - L-14 Group (n=9): sacrificed day 14 | *In vivo* (Rat model).  Population: 10-to-12-week-old male Wistar Hannover specific pathogen-free rats, N=90 (PC).  Protocol: randomised allocation, blinding of allocation not reported. Blinded examiner for alveolar bone measurements. Ligature-induced periodontitis (right and left mandibular first molars).  Measurements after 7 or 14 days of treatment (group-dependent). | Alveolar bone loss (radiographic and histological);  AL (linear distance between the ACJ and the epithelial attachment on the distal root) and inflammatory infiltrate; gene expression levels of Th17-related cytokines (*IL-6* and *IL-17*) and *RANKL* in gingivae | All intervention groups had reduced bone loss, AL, and visibly reduced inflammatory infiltrate compared to their respective L Control Group. L-TCZ2, L-TCZ4 & L-TCZ8 Groups had reduced *IL-6, IL-17* and *RANKL* expression compared to the C-7, C-14, L-7 and L-14 Groups. L-TCZ8 Group had higher *IL-6* expression compared to L-TCZ2 and L-TCZ4 Groups; time-dependent. |
| *Immunisation agents* | | | | | |
| O'Brien-Simpson *et al.* (2016);  Experiment 1: Exploratory | 50μg, intra-oral prophylactic immunisation, two vaccines spaced 30 days apart. Six groups, all with periodontitis:   - KAS1-sA1 Group (n=12): chimera immunogen of a proteolytic active site sequence and an adhesin sequence of the gingipain proteinases of *P.g.*; - KAS2-A1 Group (n=12): chimera immunogen of a proteolytic active site sequence and an adhesin sequence of the gingipain proteinases of *P.g.*; - KgpA1(759-989) Group (n=12): immunogen of an adhesin sequence of the gingipain proteinases of *P.g.;* - KgpA1(751-1056) Group (n=12): immunogen of an adhesin sequence of the gingipain proteinases of *P.g.* - FK-W50 Group (n=12): received formalin-killed (FK)-*P.g.* W50 cell-based vaccine - RgpA-Kgp Group (n=12): proteinase-adhesin complex of *P.g.* | - Non-challenged (N-C) Group (n=12): without periodontitis, no vaccine - Control Group (n=12): with periodontitis, received phosphate-buffered saline (PBS), same intra-oral schedule as intervention | *In vivo* (Mouse model).  Population: female BALB/c mice, N=96 (PC).  Protocol: randomised allocation, blinding of allocation not reported. Blinded examiners.  Infection-induced periodontitis (*P.g.* W50 on days 0 and 21).  Measurements at day 58. | Alveolar bone loss; serum levels of total IgG and IgG sub-classes (IgG1, IgG2a, IgG2b, IgG3) against FK-*P.g.* W50 cells | All intervention groups had reduced alveolar bone loss compared to Control Group. Greater reduction in alveolar bone loss for KAS2-A1 Group compared to RgpA-Kgp, FK-W50, KgpA1(759-989) and KgpA1(751-1056) Groups. KAS2-A1 selected for further experiments. Greater reduction in alveolar bone loss for KAS1-sA1 Group compared to RgpA-Kgp Group (no *p*-value). Overall, these findings indicate the KAS2-A1 vaccine was associated with greater protection against *P.g.*-induced alveolar bone loss than all other investigated immunogens except KAS1-sA1. IgG1 predominated in intervention groups pre- and post-infection. IgG2b predominated in Control Group post-infection. |
| O'Brien-Simpson *et al.* (2016);  Experiment 2: KAS2-A1 prophylactic vaccine dose | KAS2-A1 intra-oral prophylactic immunisation, formulated with aluminium hydroxide (KAS2-A1/alum) in PBS solution. Two vaccines 30 days apart. Three groups, all with periodontitis:   - KAS2-A1-50 Group (n=12): 50μg; - KAS2-A1-5 Group (n=12): 5.0μg; - KAS2-A1-0.5 Group (n=12): 0.5μg | - Non-challenged (N-C) Group (n=12): without periodontitis, no treatment - Infected Control Group (n=12): with periodontitis, no treatment - Amoxicillin Group (n=12): with periodontitis, 500µg amoxicillin per ml drinking water from day 0, no vaccine - RgpA-Kgp Group (n=12): with periodontitis, RgpA-Kgp-based control vaccine (same schedule) - PBS Group (n=12) with periodontitis, PBS-based control vaccine (same schedule) | *In vivo* (Mouse model).  Population: female BALB/c mice, N=96 (PC).  Protocol: randomised allocation, blinding of allocation not reported. Blinded examiners. Infection-induced periodontitis (*P.g.* W50 on days 0 and 21).  Measurements at day 58. | Alveolar bone loss; serum levels of total IgG, IgM and IgA as well as IgG sub-classes (IgG1, IgG2a, IgG2b, IgG3) against FK-*P.g.* W50 cells; number of *P.g.*-specific interferon (IFN)-γ- and IL-4-producing lymphocytes in maxillary gingivae; number of *P.g.*-specific IFN-γ-(represents a Th1 cell) and IL-4-producing (represents a Th2 cell) T cells in submandibular lymph nodes (SMLNs) | All KAS2-A1 doses resulted in reduced alveolar bone loss compared with the Infected Control Group.  IgG1 predominated in all intervention groups and RgpA-Kgp Group. IgG2b predominated in Infected Control and PBS Groups. No dominant IgG subclass in N-C or Amoxicillin Groups.  All intervention groups had higher numbers of IL-4- than IFN-γ-producing cells in SMLNs, indicative of a Th2-mediated immune response. In gingivae, this Th2 bias was significant for the 50μg and 0.5μg KAS2-A1 doses only. |
| O'Brien-Simpson *et al.* (2016);  Experiment 3: Therapeutic vaccination (instead of prophylactic) | KAS2-A1/alum in PBS solution, two vaccinations, days 19 and 40. Three groups, all with periodontitis:   - KAS2-A1-50 Group (n=12): 50μg - KAS2-A1-5 Group (n=12): 5.0μg - KAS2-A1-0.5 Group (n=12): 0.5μg | Without periodontitis:   - Non-challenged (N-C) Group (n=12), no treatment - Infected Control Group (n=12), no treatment - RgpA-Kgp Group (n=12): RgpA-Kgp-based control vaccine, days 19 and 40   With periodontitis:   - Amoxicillin Group (n=12): 500µg amoxicillin per ml drinking water from day 0, no vaccine - PBS Group (n=12): PBS-based control vaccine, days 19 and 40 | *In vivo* (Mouse model).  Population: female BALB/c mice, N=96 (PC).  Protocol: randomised allocation, blinding of allocation not reported. Blinded examiners. Infection-induced periodontitis (*P.g.* W50 on days 0, 2, 4 and 6).  Measurements at day 62. | Alveolar bone loss; serum levels of total IgG, IgM and IgA as well as IgG sub-classes (IgG1, IgG2a, IgG2b, IgG3) against FK-*P.g.* W50 cells; number of *P.g.*-specific IFN-γ- and IL-4-producing lymphocytes in maxillary gingivae;  Number of *P.g.*-specific IFN-γ- (represents a Th1 cell) and IL-4-producing (represents a Th2 cell) T cells in SMLNs | All KAS2-A1 intervention groups showed less alveolar bone loss compared to Infected Control Group.  IgG1 predominated in KAS2-A1-50, KAS2-A1-5 and RgpA-Kgp Groups; IgG2b predominated in Infected Control and PBS Groups. No dominant IgG subclass in KAS2-A1-0.5, N-C or Amoxicillin Groups.  All intervention groups had higher numbers of IL-4- than IFN-γ-producing cells in SMLNs, indicative of Th2-biased immune response. In gingivae, this was only significant for 50μg and 0.5μg KAS2-A1 doses. |
| O'Brien-Simpson *et al.* (2016);  Experiment 4:  Polymicrobial challenge | KAS2-A1/alum in PBS solution, 50μg, intra-oral prophylactic immunisation, two vaccinations, days 0 and 30. Two groups, with periodontitis:   - KAS2-A1 + Pg Group (n=12), infected with *P.g.* only - KAS2-A1 + Pg/Td/Tf Group (n=12), infected with *P.g.*, *T.d.* and *T.f.* | Without periodontitis:   - N-C Group (n=12), no treatment   With periodontitis:   - Control + Pg Group (n=12), with *P.g.* only, no treatment - Control + Pg/Td/Tf Group (n=12), with *P.g.*, *T.d.* and *T.f.*, no treatment | *In vivo* (Mouse model).  Population: female BALB/c mice, N=60 (PC).  Protocol: randomised allocation, blinding of allocation not reported. Blinded examiners.  Infection-induced periodontitis (as described, see intervention/control).  Measurements at day 62. | Alveolar bone loss; serum levels of total IgG and IgG sub-classes (IgG1, IgG2a, IgG2b, IgG3) against FK-*P.g.* W50 cells; number of *P.g.*-specific IFN-γ- (represents a Th1 cell) and IL-4-producing (represents a Th2 cell) T cells in SMLNs;  % gingival CD25-positive (represents an activated/antigen stimulated T cell) and CD69-positive (represents an early antigen-activated T cell) CD4+ T cells in the maxilla | Both KAS2-A1 intervention groups exhibited reduced alveolar bone less compared to their respective controls, indicative of protection against both *P.g.*-induced and *P.g.*/*T.d.*/*T.f.*-induced alveolar bone loss.  IgG1 predominated in both intervention groups, whereas IgG2b predominated in the Control + Pg and Control + Pg/Td/Tf Groups.  All intervention groups had higher numbers of IL-4-producing cells than IFN-γ-producing cells in SMLNs, and higher % of gingival CD25-positive and CD69-positive CD4+ T cells compared to N-C Group. Consistent with KAS2-A1 immunised mice exhibiting a Th2-based antigen-stimulated T cell response, while control mice exhibited a Th1-based, early antigen-activated T cell response. |
| O'Brien-Simpson *et al.* (2016);  Experiment 5: Prophylactic vaccine, topical vs intra-peritoneal injection | Purified KAS2-A1 rabbit polyclonal antibodies (KAS2-A1-pAb). Two groups, with periodontitis:   - pAb KAS2-A1 i.p. Group (n=12), intra-peritoneal injection, 500µg on day -1 - pAb KAS2-A1 topical Group (n=12), topical application, 2 x 250µg, days 0, 2, 4 and 6 (before and after infection) | Without periodontitis:   - N-C Group (n=12), no treatment   With periodontitis:   - Infected Control Group (n=12), no treatment - Amoxicillin Group (n=12), 500µg amoxicillin per ml drinking water from day 0, no vaccine - pAb PBS/alum i.p. Group (n=12), intra-peritoneal injection, 500µg, non-specific purified rabbit polyclonal antibodies on day -1 - pAb PBS/alum topical Group (n=12), topical PBS/alum-pAb, 2 x 250µg, days 0, 2, 4 and 6 (before and after infection) | *In vivo* (Mouse model).  Population: female BALB/c mice, N=84 (PC).  Protocol: randomised allocation, blinding of allocation not stated. Blinded examiners. Infection-induced periodontitis (*P.g.).*  Measurements at day 62. | Alveolar bone loss | Both intervention groups had reduced alveolar bone loss compared to the Infected Control Group and their respective controls.  Indicative that passive prophylactic immunisation with KAS2-A1 antibodies protects against *P.g.*-induced alveolar bone loss, irrespective of whether antibodies are delivered topically or intra-peritoneal injection, and beyond effects achieved with non-specific antibodies. |
| O'Brien-Simpson *et al.* (2016);  Experiment 6: Therapeutic vaccine, topical vs intra-peritoneal injection | Purified KAS2-A1 rabbit polyclonal antibodies (KAS2-A1-pAb). Two groups, with periodontitis,   - pAb KAS2-A1 i.p. Group (n=12), intra-peritoneal injection, 500µg, 19 days after infection - pAb KAS2-A1 topical Group (n=12), topical application, 4x 500µg, from day 19 | Without periodontitis:   - N-C Group (n=12), no treatment   With periodontitis:   - Infected Control Group (n=12), no treatment - Amoxicillin Group (n=12), 500µg amoxicillin per ml drinking water from day 0, no vaccine - pAb PBS/alum i.p. Group (n=12): intra-peritoneal injection, non-specific purified rabbit polyclonal antibodies - pAb PBS/alum topical Group (n=12), topical | *In vivo* (Mouse model).  Population: female BALB/c mice, N=72 (PC).  Protocol: randomised allocation, blinding of allocation not reported. Blinded examiners. Infection-induced periodontitis (*P.g.).*  Measurements at day 62. | Alveolar bone loss | Both KAS2-A1-pAb intervention groups had less alveolar bone loss than the Infected Control Group and their respective controls. Indicative that passive therapeutic immunisation with KAS2-A1 antibodies protects against *P.g.*-induced alveolar bone loss, irrespective of whether antibodies are delivered topically or via intra-peritoneal injection, and beyond effects achieved with non-specific antibodies. |
| Sugano *et al.* (2009) | Anti-*Porphyromonas gingivalis* egg yolk antibody against gingipains (IgY-GP) (n=18). Oral administration of 50mg tablet (instructed to be sucked for as long as possible without chewing or swallowing). | Oral administration of tablet containing non-immunised egg-yolk antibody, (n=16), same instruction as intervention group. | Randomised controlled trial.  Population: adult patients with untreated periodontitis, N=34 (NPC).  Duration: 12 weeks.  Protocol: double-blinded. All received full-mouth SRP. | PPD; %BOP; sub-gingival number and level of *P.g.* in deepest periodontal pocket | IgY-GP group demonstrated greater reduction in PPD, no difference for %BOP. Intervention group showed reduction in both number and level of *P.g* in deepest periodontal pocket. |
| *Plant-derived compounds* | | | | | |
| Abidi *et al.* (2022) | - Cannabigerol, - Cannabidiol, or - Cannabidivarin   Each administered at 1μg/ml in serum-free Basal Fibroblast Medium (BFM) growth medium containing 1ng/ml of IL-1β. | Serum-free BFM growth medium containing 1ng/ml of IL-1β. | *In vitro* (Human cell line).  Cell type: human gingival fibroblasts (HGFs) from one healthy 28-year-old Caucasian male (NPC).  Protocol: non-blinded.  Measurements after 24 hours. | Prostaglandin E2 (PGE2); cytokine expression: IL-2, IL-4, IL-6, IL-8, IL-10, IL-12, IL-13, TNF-α and IL-1β | Cannabidiol decreased IFN-γ, TNF-α and IL-2 expression, and increased IL-6, IL-10 and IL-12 expression.  Cannabidivarin decreased PGE2, IFN-γ, TNF-α, IL-2, IL-4 and IL-13 expression, and increased IL-6, IL-10 and IL-12 expression.  Cannabigerol decreased PGE2, IFN-γ, TNF-α, IL-2 and IL-4 expression, and increased IL-6, IL-10 and IL-12 expression. |
| Cafferata *et al.* (2021) | Boldine (bioactive compound extracted from the Peumus boldus Molina species of boldo tree). Daily oral dosing boldine in 100μL PBS for 15 days. Three groups, with periodontitis:   - Group C (n=8): 10mg/kg (low) - Group D (n=8): 20mg/kg (medium) - Group E (n=8): 40mg/kg (high) | - Group A (n=8): without periodontitis, no oral dosing - Group B (n=8): with periodontitis, daily oral dosing of 100μL PBS for 15 days | *In vivo* (Mouse model).  Population: 7-week-old C57BL/6 mice, N=40 (NPC).  Protocol: randomised allocation, blinding of allocation not reported. Blinded examiner. Ligature induced periodontitis (maxillary second molars).  Measurements at day 15. | Alveolar bone loss (ACJ-ABC distance); number of TRAP-positive-cells;  GCF levels of receptor activator of nuclear factor kappa-B ligand (RANKL) and osteoprotegerin (OPG); inflammatory cell counts (lymphocytes, neutrophils and macrophage-like cells);  periodontal tissue expression of RANKL, OPG, Th17 & Treg-associated transcription factors (retinoic acid-related orphan receptor gamma t [RORγt] and forkhead box P3 [Foxp3]), Th17 cytokines (IL-6, IL-17A and IL-23), Treg cytokines (IL-10, IL-35 and transforming growth factor (TGF)-1β), and other cytokines (IL-1β, IL-12, IFN-γ and TNF-α);  Th17 and Treg cells in cervical lymph nodes (CLNs) | Boldine treatment (Groups C, D, E) associated with reduced alveolar bone resorption compared with Group A; evidence of dose-dependent effect.  At all doses, the number of TRAP-positive cells were reduced when compared to Group B.  Compared with Group B, RANKL expression was lowered at all doses, OPG was increased at medium and high doses, and RANKL/OPG ratio was reduced; dose-dependent.  Compared with Group B, mRNA expression was reduced for: IL-1β, IL-12, IFN-γ, TNF-α, IL-6, IL-17A, IL-23 and RORγt; dose-dependent. Meanwhile, IL-10, IL-35, TGF-1β and Foxp3 were increased; dose-dependent  In CLNs, Th17 cells were reduced and Treg cells were increased in number and proportion.  Visually fewer inflammatory cells (no *p*-value). |
| Kim *et al.* (2019);  Experiment 1: Cell line | 6-shogaol (a bioactive compound in dried ginger) at 2.5-10µM added to culture medium containing 20ng/ml macrophage colony-stimulating factor (M-CSF) and 100ng/ml RANKL. | Culture medium containing 20ng/ml M-CSF and 100ng/ml RANKL only. | *In vitro* (Mouse cell line).  Cell type: bone marrow macrophages (BMMs) from tibia and femur of 5-week-old C57BL/6 mice (NPC).  Protocol: blinding protocol not reported.  Measurements at days 2-7. | BMM differentiation into osteoclasts (number of TRAP-positive cells); subsequent osteoclast function (formation of actin rings, extent of dentine resorption); osteoclast intracellular signalling pathways | 6-shogaol decreased number of TRAP-positive cells in a concentration-dependent manner. At 7.5µM, decreased actin ring formation and dentine resorption (no *p*-values).  Expression of various osteoclast marker & fusion genes, signalling proteins, Ca^2+^ concentrations, and reactive oxygen species (ROS) levels were all reduced. |
| Kim *et al.* (2019);  Experiment 2: Mouse model | 6-shogaol (n=14; n=7): with periodontitis. Intra-peritoneal injection of 10mg/kg on days 1, 4 and 7. | Intra-peritoneal injection of vehicle on days 1, 4 and 7 (n=14; n=7): with periodontitis. | *In vivo* (Mouse model).  Population: 8-week-old male C57BL/6 mice (NPC)  Protocol: randomised allocation, blinding of allocation not reported. Blinded examiners. Ligature-induced periodontitis (left maxillary second molars).  Measurements after day 10. | Alveolar bone loss (ACJ-ABC distance, % bone area and % eroded surfaces);  Number of osteoclasts (TRAP-positive cells), macrophages (F4/80-positive cells), neutrophils (Ly6G-positive cells) and T cells (CD3-positive cells); expression of IL-1β, IL-6 and TNF-α | Reduced alveolar bone loss compared with vehicle controls. Reduced number of TRAP-, F4/80- and Ly6G-positive cells. No reduction in CD3-positive cells. Lower expression of IL-1β and TNF-α, non-significant for IL-6. |
| Leguizamon *et al.* (2022);  Experiment 1: *in vitro* | CsinCPI-2 (cysteine peptidase inhibitor derived from *Citrus sinensis* orange tree), 2.5-100µM in 2ml culture medium with 30ng/ml recombinant murine M-CSF and 4ng/ml recombinant murine RANKL, 1-4 days. | 2ml culture, 1-4 days:   - Group A: Medium + M-CSF only - Group B: Medium + M-CSF + RANKL | *In vitro* (Mouse cell line).  Cell type: BMMs from femur and tibia of 4–6-week-old male mice, N= unreported (NPC).  Protocol: randomisation/blinding protocol not stated.  Measurements after 1-4 days. | TRAP-positive osteoclast differentiation; subsequent osteoclast activity (formation of actin rings); RANKL expression of osteoclast phenotypic genes (*Ctsk, Acp5, Calcr* and *Nfatc1*) | Decreased number of TRAP-positive cells; time- and concentration-dependent. Visibly reduced actin ring formation (no *p*-value) and RANKL-induced expression of *Calcr*, *Acp5*, *Ctsk* and *Nfatc1.* |
| Leguizamon *et al.* (2022);  Experiment 2: *in vivo* | CsinCPI-2, n=8, with periodontitis, daily intra-peritoneal injections, 0.8μg/gram bodyweight, in 200µL of PBS. | - Group A (n=8): with periodontitis daily 200µL PBS injection - Group B (n=8): without periodontitis, received intervention at same dose [classified as controls] | *In vivo* (Mouse model).  Population: 8-week-old male C57BL/6J mice, N=24 (NPC).  Duration: 15 days.  Protocol: randomised allocation, blinding of allocation not reported. Blinded examiners. Ligature-induced periodontitis (left & right maxillary first molars). | Alveolar bone height (ACJ-ABC distance), volume and density; proportion of fibroblasts, inflammatory cells, vascular structures and extracellular matrix (ECM) in periodontal tissue; number of TRAP-positive cells; number of CD45- (marker of leukocyte), CD3- (marker of T cell), MAC387- (marker of macrophage) and IL-1β-positive cells; mRNA expression of IL-1β in gingival tissue | Intervention group compared to Group A: reduced ACJ-ABC distance, and increased bone volume and density; reduced number of TRAP-positive cells and inflammatory cells, including reduced number of CD3-, CD45- and MAC387-positive cells; reduced IL-1β expression; increased proportion of fibroblasts. |
| *Anti-hyperglycaemic drugs* | | | | | |
| Kawahara *et al.* (2020);  Experiment 1: *in vitro* | Seven agents:   - MCC950 (an NLRP3 inhibitor) at 10nM, 100nM, 1µM, and 10µM - z-YVAD-FMK (a capase-1 inhibitor) at 0.1µM, 0.5µM, 1µM, and 2µM - metformin at 0.5mM, 1mM, 2.5mM, and 5mM - glyburide or tolbutamide or gliclazide or glimepiride, all at 25µM, 50µM, and 100µM | - Cells not stimulated with a bacterium or lipopolysaccharide (LPS) + nigericin - Bacterium- or LPS + nigericin-stimulated cells treated with DMSO - Bacterium- or LPS + nigericin-stimulated cells treated with PBS only | *In vitro* (Human cell line).  Cell type: differentiated THP-1 (human monocyte) macrophage-like cells (NPC).  Protocol: randomisation/(examiner) blinding protocol not reported. All intervention and some control stimulated with one of: *P.g.*, *A.a.*, *F.n*., *E.c.* (a Gram-negative reference), *A.v.* (a Gram-positive reference), or LPS + nigericin (an NLRP3 stimulator). Exposure time 45 minutes or 24 hours. | Comparison of cell expression levels of IL-1β release by stimulated THP-1 cells; expression levels of caspase-1 (visual assessment, western blots) | IL-1β release was reduced in cells pre-treated with MCC950, z-YVAD-FMK or glyburide (all bacteria- and concentration-dependent) and cells treated with glimepiride (concentration-dependent).  Greater IL-1β release in cells treated with tolbutamide and gliclazide (both bacteria- and concentration-dependent).  Caspase-1 levels were visibly reduced in cells treated with glyburide (no *p*-value).  No changes reported for metformin. |
| Kawahara *et al.* (2020);  Experiment 2: *in vivo* | Glyburide, daily oral administration of 20mg/kg body weight in 20% ethanol for 4 days.   - PBS + intervention (n=8) - *P.g.* + intervention (n=8) - *A.a.* + intervention (n=8) - *E.c.* + intervention (n=8) | Ethanol only (20%), daily oral administration for 4 days.   - PBS + control group (n=8) - *P.g.* + control group (n=8) - *A.a.* + control group (n=8) - *E.c.* + control group (n=8) | *In vivo* (Mouse model).  Population: 7-week-old male Lewis rats (Oriental Yeast), N=64 (NPC).  Duration: 4 days after initial infection.  Protocol: randomisation/(examiner) blinding protocol not reported. Infection-induced periodontitis (*P.g.* or *A.a.*, *E.c.* used as a Gram-negative reference, PBS used as a sham injection). | Infiltrated inflammatory cell numbers in connective tissue above alveolar bone crest; alveolar bone resorption (ACJ-ABC distance); number of TRAP-positive cells in the periodontal tissues and alveolar bone surface; IL-1β expression levels in gingivae | Reduced alveolar bone loss in the *P.g.* + intervention, *A.a.* + intervention, and *E.c.* + intervention Groups compared to their respective control.  Reduced number of inflammatory cells and TRAP-positive cells in the *A.a.* + intervention and *E.c.* + intervention Groups compared to their respective control.  Visually reduced IL-1β expression (no *p*-value). |
| Zhou *et al.* (2019);  Experiment 1: Cell line | Metformin, 1mM, 5mM or 10mM in high-glucose Dulbecco’s modified Eagle medium (DMEM) containing 4,500mg/L glucose (to induce diabetes), with periodontitis. | High-glucose DMEM containing 4,500mg/L glucose, with or without periodontitis. | *In vitro* (Mouse cell line).  Cell type: Raw 264.7 (murine) cells (NPC).  Protocol: randomisation/(examiner) blinding not reported. Infection-induced periodontitis (100ng/ml *P.g.*-derived LPS). | Cell expression levels of Nek7, NLRP3, caspase-1 and mammalian target of rapamycin (mTOR) proteins | Lowered expression of Nek7, NLRP3, caspase-1 and mTOR; dose-dependent. |
| Zhou *et al.* (2019);  Experiment 2: Mouse model | Group DPM (n=10): with periodontitis and diabetes mellitus, daily 200mg per kg bodyweight oral metformin, from week 4 for 10 weeks. | Sterile water, volume matched to intervention. Three groups:   - Group N (n=10): normal controls - Group D (n=10): with diabetes mellitus - Group DP (n=10): with diabetes mellitus & periodontitis | *In vivo* (Mouse model).  Population: 4-week-old male C57BL/6 wild-type mice, N=40 (NPC).  Duration: 16 weeks.  Protocol: randomised, blinded allocation. Examiner blinding not reported. Infection-induced periodontitis (*P.g.*). | Alveolar bone loss; attachment loss (ACJ-ABC distance); tooth displacement angle; serum concentrations of IL-1β; presence of macrophages (CD206-positive cells) in the maxillae; expression of NIMA-related kinase 7 (Nek7), Nucleotide-binding oligomerisation domain-like receptor family pyrin domain containing 3 (NLRP3), caspase-1 and mTOR proteins | Reduced alveolar bone loss, attachment loss, serum IL-1β levels, and expression of Nek7, NLRP3, caspase-1 & mTOR compared to Group DP.  Higher average tooth displacement angle compared to Group DP, indicating less severe displacement. No difference in average displacement angle compared to Group N, indicating metformin-treated mice share a similar degree of displacement to healthy controls.  Visibly less macrophage infiltration compared to Group DP, but higher compared to Group N. |
| *Vitamins, their derivatives and/or their metabolites* | | | | | |
| Tsutsumi *et al.* (2011) | L-ascorbic acid 2-phosphate magnesium salt [APM] (a derivative of vitamin C), 50μM in Dulbecco’s modified Eagle’s medium (DMEM) containing 1% foetal calf serum (FCS). | Two groups:  No treatment Group;  AsANa Group: 50μM L-ascorbic acid sodium salt (AsANa) in DMEM containing 1% FCS  (AsANa equivalent to standard vitamin C). | *In vitro* (Human cell lines).  Cell type: human gingival fibroblast cell lines (HGF-1, HGF-2 from N=6 (n=4) male healthy 12-to-18-year-old donors; Gin-1 commercial cell line) (NPC).  Protocol: randomisation/(examiner) blinding protocol not reported. Exposure times varied. | Type I collagen synthesis; mRNA expression of type I collagen; hydrogen peroxide- or TNF-α-induced intracellular ROS synthesis; TNF-α-induced IL-8 production | Findings mainly reported for HGF-1 cell line.  Greater synthesis and mRNA expression of type I collagen compared to both controls; time-dependent.  Reduced ROS synthesis compared to both controls; time-dependent compared to AsANa Group only.  Reduced TNF-α induced ROS and IL-8 expression compared to both controls.  [The AsANa Group demonstrated increased type I collagen synthesis and reduced ROS synthesis (time-dependent) compared to the No treatment Group] |
| Wang *et al.* (2014) | All-*trans* retinoic acid [ATRA] (an active metabolite of vitamin A), n=4, with periodontitis. Oral gavage with 25μg ATRA per gram of bodyweight “every other day”. | Two groups:  Placebo Group (n=4): with periodontitis, received placebo carboxymethylcellulose (CMC);  Sham Group (n=4): without periodontitis, no treatment. | *In vivo* (Mouse model).  Population: 7-week-old female C57BL/6 mice, N=12 (NPC).  Duration: experiment end at day 35.  Protocol: randomised allocation, blinding of allocation not reported. Examiner blinding not reported. Infection-induced periodontitis (*P.g).* | Alveolar bone loss; inflammatory cell infiltration in samples from maxilla; number and proportion (%) of CD4+ T helper cell subsets in CLNs and spleen; mRNA expression levels of Th17/Treg cell-related cytokine (IL-17a, IL-10 and TGF-1β) in CLN and gingival tissue | Compared to Placebo Group:   - Reduced alveolar bone loss - Fewer CD4+ T cells in CLNs, with reduced % of Th17 cells and higher % of Treg cells, and fewer RANKL+ CD4+ T cells in the CLN and spleen - Lower expression of IL-17a, and higher expression of IL-10, and TGF-1β   Compared to Sham Group: no difference in alveolar bone loss, and higher RANKL+ CD4+ T cells in CLN.  Histologically, visibly less inflammatory cell infiltration and attachment loss, with no apparent alveolar bone resorption, compared to Placebo Group (no *p*-values). |
| Yu, Valerio and Bielawski (2013) | Fenretinide (a synthetic analogue of vitamin A), incubation with 2.5-5μM dissolved in ethanol | Incubation in ethanol | *In vitro* (Mouse cell line).  Cell type: mouse monocyte/ macrophage (Raw 264.7) cells (NPC).  Protocol: blinding protocol not reported. Exposure time varied from 4-16 hours. | Production of sphingolipids (dhSph, dhCer, ceramide, sphingosine, sphingosine-1-phosphate and sphingomyelin); mRNA expression of IL-1β, IL-6, TNF-α and COX-2; protein expression of IL-1β, IL-6, TNF-α and PGE2; phosphorylation (p-) status of STAT1, PI3K, Akt, PKC, IκBα, NF-κB p65, ERK, JNK and p38 signalling proteins | Increased dhSpH and dhCer synthesis.  Reduced synthesis of ceramide, sphingosine and sphingomyelin.  Reduced mRNA levels of IL-1β, IL-6 & COX-2 (dose-dependent for IL-6 and Cox-2), and protein levels of IL-1β, IL-6 & PGE2.  Lower levels of p-STAT1, p-PI3K, p-Akt, p-PKC, p-IκBα and p-NF-κB p65; time-dependent. |
| *Chemokine-based agents* | | | | | |
| Glowacki *et al.* (2013);  Experiment 1: *P.g.* induced periodontitis | C-C motif chemokine ligand 22 (CCL22).  CCL22 Group: with periodontitis, gingival injection of CCL22-loaded polylactic-co-glycolic acid (PLGA) microparticles suspended in PBS with 2% CMC, 25 mg/ml, on day -1 (before infection), 20 and 40 | Two groups:  Blank Group: with periodontitis, received blank PLGA microparticles;  No Pg Group: without periodontitis (treatment not reported). | *In vivo* (Mouse model).  Population: male BALB/c mice, N=unreported (NPC).  Protocol: randomisation/(examiner) blinding protocol not reported. Infection-induced periodontitis (*P.g.*).  Measurements at 30 days after infection. | Alveolar bone loss (ACJ-ABC area); Foxp3-positive cells in periodontal tissues | Reduced alveolar bone loss compared to Blank Group. Foxp3-positive cells were only histologically detectable in tissue sections from the CCL22 Group, indicating CCL22 therapy is associated with influx of Treg cells into periodontal tissue. |
| Glowacki *et al.* (2013);  Experiment 2: *A.a.* induced periodontitis | CCL22 Group: with periodontitis,  gingival injection of CCL22-loaded PLGA microparticles suspended in PBS with 2% CMC, 25 mg/ml, on days -1 (i.e. 1 day before infection), 10 and 20 . | Three groups:  Untreated Group: with periodontitis, no treatment;  Blank Group: with periodontitis, received blank PLGA microparticles;    No Aa Group: without periodontitis (treatment unclear). | *In vivo* (Mouse model).  Population: male wild-type C57BL/6 mice, N= unreported (NPC).  Protocol: randomisation/(examiner) blinding protocol not reported. Infection-induced periodontitis (*A.a.*).  Measurements at 30 days after infection. | Alveolar bone loss (ACJ-ABC area); inflammatory cell infiltrate; mRNA expression levels of Foxp3, anti-inflammatory cytokines (IL-10, TGF-β), CTLA-4 (anti-inflammatory protein), Th2 cell-associated markers (IL-4, CCL22), pro-inflammatory cytokines (IL-1β, IFN-γ, TNFα, IL-17), IL-2, IL-12, CCR4 (receptor for CCL22 which is highly expressed by Treg cells), bone pro-regenerative markers (BMPs, RUNX2, COL1A1, COL1A2), alkaline phosphatase, Receptor Activator of Nuclear Factor Kappa-B, RANKL, OPG, TIMPs and MMPs; protein levels of: TGF-β, IL-10, CTLA-4 and TNF;  Concentration of IL-10, TGF-β, TNF, CTLA-4 and RANKL | Compared to both controls:   - Reduced alveolar bone loss and inflammatory cell infiltrate - Reduced protein levels of TNF and RANKL - Reduced mRNA expression of RANKL, IL-1β, IFN-γ, TNF-α, IL-17, IL-2, IL-12 and CCR4 (no *p*-values for IL-2, IL-12 and CCR4) - Greater protein levels of TGF-β, IL-10 and CTLA-4 - Greater mRNA expression of Foxp3, IL-10, TGF-β and CTLA-4 - Greater expression of BMP7, RUNX2 and COL1A1   Compared to Untreated Group:   - Greater expression of DMP-1, TIMP-1, TIMP-3 and COL1A2 (no *p*-values except for DMP-1) - Reduced mRNA expression of Receptor Activator of Nuclear Factor Kappa-B, MMP-2, MMP-8 and MMP-9 (no *p*-values) |
| Glowacki *et al.* (2013);  Experiment 3: Ligature-induced periodontitis | CCL22 Group (n=3): with periodontitis, deposition of ~2-4mg of dry CCL22-loaded PLGA microparticles into periodontal pockets immediately after ligature placement and 4 weeks later. | Two groups:  Blank Group (n=3): with periodontitis, received blank PLGA microparticles;  Untreated Group (n=3): with periodontitis, no treatment. | *In vivo* (Dog model).  Population: female beagle dogs, N=9 (NPC).  Duration: 8 weeks.  Protocol: randomisation/(examiner) blinding protocol not reported. Ligature-induced periodontitis (mandibular teeth). Received SRP then twice-daily toothbrushing before ligature placement.  Measurements at 0, 4 and 8 weeks after ligature placement. | PPD; %BOP; alveolar bone loss (ACJ-ABC distance) | Reduced alveolar bone loss compared to both controls.  Reduced PPD compared to Untreated Group (time-dependent) and Blank Group.  Lower %BOP compared to Blank Group; time-dependent. |
| *Polymer-loaded nanofibres* | | | | | |
| He *et al.* (2024);  Experiment 1: Mouse model | Poly(ɛ-caprolactone), sub-gingival insertion of nanofibers into periodontal pockets at baseline only:   - PLN Group (n=1): poly(ɛ-caprolactone)-loaded nanofibers with highly-oriented periodic lamellae structure (PLN) - PCL Group (n=1): poly(ɛ-caprolactone)-loaded nanofibers without periodic lamellae structure (PCL) | Control Group (n=1): injected with saline solution, no treatment. | *In vivo* (Mouse model).  Population: 8-week-old male C57BL/6J mice, N=3 (NPC).  Duration: experiment end at 1 and 3 weeks after bone defect creation surgery.  Protocol: randomisation/blinding protocol not reported. Ligature-induced periodontitis (maxillary second molar). | Alveolar bone quantity (ACJ-ABC distance, bone volume/tissue volume [BV/TV], trabecular separation [Tb.Sp], trabecular number); IL-1β tissue expression levels | PLN Group demonstrated:   - Greater BV/TV compared to the PLC and Control Groups - Reduced ACJ-ABC, Tb.Sp (time-dependent) and IL-1β expression compared to the PLC and Control Groups, and lower trabecular number compared to the Control Group (time-dependent)   Overall, PLN Group had visibly lesser alveolar bone loss than PCL and Control Groups.  No findings of significance were reported for PCL Group. |
| He *et al.* (2024);  Experiment 2: Macrophage cell line | Poly(ɛ-caprolactone):   - PLN Group - PCL Group   See Experiment 1 for details. | Control Group: no treatment. | *In vitro* (Mouse cell lines).  Cell type: Raw 264.7 cells from American Type Culture Collection (NPC).  Protocol: randomisation/(examiner) blinding protocol not reported. Infection-induced periodontitis (500ng/ml LPS). Exposure time varied. | M1-phenotype macrophage count (CCR7- or CD86-expressing cell);  M2-phenotype macrophage count (CD206-expressing cell); expression levels of M1 phenotype-related genes (*IL-1β, IL-6, TNF-α, CD86*) and proteins (IL-1β, TNF-α, CCR7); expression levels of M2 phenotype-related genes (*TGF-β, CD206*) and proteins (TGF-β, CD206); Raw264.7-stimulated migration of bone marrow stromal cells (BMSCs) | PCL Group demonstrated reduced expression of *IL-6* gene and TNF-α compared to Control Group.  PLN Group demonstrated:   - Fewer CD86- and CCR7-expressing cells and more CD206-expressing cells compared to PCL and Control Groups - Reduced expression of *IL-1β, IL-6* & *TNF-α* genes and TNF-α protein compared to PCL and Control Groups. Reduced expression of *CD86* gene and CCR7 & IL-1β proteins compared to Control Group - Higher expression of *CD206* gene and CD206 & TGF-β proteins compared to PCL and Control Groups. Higher expression of *TGF-β* gene compared to Control Group - Greater migration rate of BMSCs compared to PCL and Control Groups |
| He *et al.* (2024);  Experiment 3: Bone marrow stromal cell (BMSC) line | Poly(ɛ-caprolactone):   - PLN Group - PCL Group   See Experiment 1 for details. | Control Group: no treatment. | *In vitro* (Mouse cell line).  Cell type: BMSCs from 3-week-old male C57BL/6J mice (NPC).  Protocol: same as Experiment 2. | Cell migration volume; expression levels of Runx2 (osteogenic protein) | PLN Group demonstrated greater cell migration and Runx2 expression compared to PCL and Control Groups.  PCL Group demonstrated greater cell migration than the Control Group. |
| *Tricyclic antidepressants* | | | | | |
| Branco-de-Almeida *et al.* (2020) | Desipramine Group (n=20): with periodontitis, daily oral gavage, 20mg/kg desipramine in saline. | Daily oral gavage with saline:   - Control Group (n=20): without periodontitis - Ligature Group (n=20): with periodontitis | *In vivo* (Rat model).  Population: male Wistar rats, N=60 (PC).  Protocol: randomised allocation, blinding of allocation not reported. Blinded examiner. Ligature-induced periodontitis (mandibular first molar).  Gingival samples taken at day 3, bone samples at day 15. | Alveolar bone loss;  collagen fibre content in connective tissue;  gingival tissue mRNA expression of IL-1β, iNOS, COX-2, MMP-9 and TIMP-1; MMP-9 protein activity in the gingivae | Reduced alveolar bone loss compared to Ligature Group, comparable bone loss to Control Group.  Reduced collagen fibre content compared to Control Group.  Greater MMP-9, IL-1β, iNOS & COX-2 expression and MMP-9/TIMP-1 ratio compared to Control Group.  Lower MMP-9 activity and lower IL-1β, iNOS, COX-2 and TIMP-1 expression compared to Ligature Group. |
| Breivik *et al.* (2006) | Tianeptine-treated, olfactory bulbectomized (OB) Group (n=10): OB to induce depression, twice daily intra-peritoneal injections, 10mg/kg dissolved in saline. | Twice daily intra-peritoneal injections of saline:   - Saline-treated OB Group (n=10): OB to induce depression - Control Group (n=10): sham-operated rats | *In vivo* (Rat model).  Population: male Wistar rats, N=30 (NPC).  Duration: experiment end at day 62.  Protocol: randomised allocation, blinding of allocation not reported. Blinded examiner for alveolar bone measurements. Ligature-induced  periodontitis (right maxillary second molar). | Alveolar bone loss; serum levels of corticosterone, TNF-α, IL-10 and TGF-1β | Reduced alveolar bone loss compared to Saline-treated OB Group.  Higher serum TNF-α and TGF-1β compared to Saline-treated OB Group.  Higher serum TGF-1β and IL-10 compared to Control Group. |
| *microRNA-based agents* | | | | | |
| Luo *et al.* (2023);  Experiment 1: Cell line | microRNA-126 [miR-126] (a macrophage-associated anti-inflammatory microRNA molecule). Local injection into periodontal tissue. Two groups:  CXCR4-miR126-Exo Group: injection of C-X-C chemokine receptor type 4 (CXCR4)-overexpressing exosome loaded with miR-126;  miR126-Exo Group: injection of non-CXCR4-overexpressing exosome loaded with miR-126. | Two groups:  Ctrl-Exo Group: local injection of non-CXCR4-overexpressing exosome loaded with negative control into periodontal tissue;  CXCR4-Exo Group: treatment with CXCR4-overpressing exosome loaded with negative control. | *In vitro* (Human cell lines).  Cell type/relevant population: human monocytic cell (THP-1)-derived M1 macrophages. NPC.  Duration: 48 hours.  Protocol: randomised/blinding protocol not reported for THP-1 experiments. Infection-induced periodontitis (1µg/ml LPS & 50ng/ml IFN-γ). | Expression levels of pro-inflammatory (*IL-1α, IL-6, TNF-α*) and anti-inflammatory (*IL-4, IL-10*) genes; synthesis and secretion levels of pro-inflammatory (IL-1α, IL-6, TNF-α) and anti-inflammatory (IL-4, IL-10) proteins | The miR126-Exo Group demonstrated:   - Reduced gene expression of *IL-1α*, *IL-6* and *TNF-α* and increased gene expression of *IL-4* and *IL-10* compared to Ctrl-Exo Group - Increased IL-4 synthesis and increased IL-4 and IL-10 secretion compared to Ctrl-Exo Group   The CXCR4-miR126-Exo group demonstrated:   - Reduced gene expression of *IL-6* and *TNF-α* compared to miR126-Exo Group and both controls, and reduced *IL-1α* gene expression compared to the controls. Increased gene expression of *IL-10* and *IL-4* compared to miR126-Exo Group and both controls - Reduced synthesis of IL-6 and TNF-α compared to miR126-Exo Group and both controls. Reduced synthesis and secretion of IL-1α compared to the controls - Greater synthesis of IL-10 compared to miR126-Exo Group and both controls, and greater secretion of IL-10 compared to the controls. Greater synthesis of IL-4 compared to miR126-Exo and Ctrl-Exo Groups, and greater secretion of IL-4 compared to miR126-Exo Group and both controls |
| Luo *et al.* (2023);  Experiment 2: Rat model | miR-126 (a macrophage-associated anti-inflammatory microRNA molecule). Intra-peritoneal injection, 300µg, at baseline only. Two groups:  CXCR4-miR126-Exo Group: injection of CXCR4-overexpressing exosome loaded with miR-126;  miR126-Exo Group: injection of non-CXCR4-overexpressing exosome loaded with miR-126. | Intra-peritoneal injection, 300µg, at baseline only. Two groups:  Ctrl-Exo Group: injection of non-CXCR4-overexpressing exosome loaded with negative control;  Blank-Ctrl Group: injection of PBS. | *In vivo* (rat model).  Population: 8-week-old male Sprague-Dawley rats, N=not reported (NPC).  Duration: 6 weeks.  Protocol: randomised allocation, blinding of allocation not reported. Examiner blinding not reported. Ligature-induced periodontitis (maxillary second molars).  Measurements at 2 weeks after initiating treatment. | Alveolar bone loss (ACJ-ABC distance); gene expression of pro-inflammatory cytokines (*IL-1α, IL-6, TNF-α*) and anti-inflammatory cytokines (*IL-4, IL-10*) in gingival tissue; osteoclast (TRAP-positive cell) count; M1 phenotype macrophage count (CD68-positive cell); M2 phenotype macrophage count (CD206-positive cell); CD206-positive/CD68-positive cell ratio | The miR126-Exo Group demonstrated:   - Reduced alveolar bone loss compared to the controls - Reduced gene expression of *IL-1α* and *TNF-α* compared to both controls and reduced gene expression of *IL-6* compared to Blank-Ctrl Group. Increased gene expression of *IL-4* and *IL-10* compared to both controls - Lower osteoclast count compared to both controls and lower M1 macrophage count compared to Blank-Ctrl Group. Higher M2 macrophage count and M2/M1 macrophage ratio than both controls   The CXCR4-miR126-Exo Group demonstrated:   - Reduced alveolar bone loss compared to miR126-Exo Group and both controls - Reduced gene expression of *IL-1α* and greater expression of *IL-4* & *IL-10* compared to miR126-Exo Group and both controls. Reduced expression of *IL-6* and *TNF-α* compared to the controls - Lower osteoclast & M1 macrophage count, and higher M2 macrophage count & M2/M1 macrophage ratio compared to miR126-Exo Group and both controls |
| *Beta-glucans* | | | | | |
| Breivik *et al.* (2005) | Purified beta-1,3/1,6-glucan [β-1,3/1,6-glucan] (a carbohydrate polymer isolated from the cell wall of *Saccharomyces cerevisiae*).  β-glucan Group (n=15): daily administration of β-1,3/1,6-glucan in drinking water, 10mg/kg. | Control Group (n=15): daily provision of tap water only. | *In vivo* (Rat model).  Population: male Wistar rats, N=30 (NPC).  Duration: Experiment end 35 days after ligature placement.  Protocol: randomised allocation, blinding of allocation not reported. Blinded examiner for radiographic measurements. Ligature-induced PD (right maxillary second molar). | Alveolar bone loss (distance between ACJ and the most coronal alveolar bone on mesial and distal surfaces of maxillary second molars);  serum levels of corticosterone, IL-10, TGF-1β and TNF-α | Lost during experiment: n=4 (n=2 per group).  Reduced bone loss.  Higher serum corticosterone and TGF-1β levels. |
| *Strontium ranelate* | | | | | |
| Karakan *et al.* (2017) | Strontium ranelate (SR), daily gastric gavage. Three groups with periodontitis:  SR300 Group (n=8): 300mg/kg;  SR625 Group (n=8): 625mg/kg;  SR900 Group (n=8): 900mg/kg. | Two groups:  Control Group (n=8): without periodontitis, daily gastric gavage with saline;  Ligated Group (n=8): with periodontitis, daily gastric gavage with saline. | *In vivo* (Rat model).  Population: male Wistar rats, N=40 (NPC).  Duration = experiment end on day 12.  Protocol: randomised allocation, blinding of allocation not reported. Blinded examiner for alveolar bone measurements. Ligature-induced periodontitis (mandibular right first molar). | Alveolar bone loss (ACJ-ABC distance);  Serum levels of IL-1β, OPG and BALP; osteoblast and osteoclast count; inflammatory cell infiltrate; RANKL activity (number of RANKL-positive osteoblastic cells) | Lost during experiment: n=1 (SR900 group).  Greater alveolar bone loss compared to Control Group at all doses. Only the SR900 Group had reduced alveolar bone loss compared to Ligated Group.  SR300 and SR900 Groups had reduced osteoclast numbers compared to Ligated Group.  SR625 and SR900 Groups had higher osteoblast numbers compared to Ligated Group; dose-dependent effect.  Greater inflammatory infiltrate compared to Control Group at all doses.  SR300 and SR625 Groups had higher RANKL activity compared to Control Group. |
| *Growth factors* | | | | | |
| Liu *et al.* (2024) | Progranulin (PGRN)-loaded gelatin methacrylol (GelMA) [PGRN/GelMA] complex. One group:  SRP + PGRN/GelMA Group (n=8 teeth from n=8 dogs): SRP once followed by weekly sub-gingival delivery of 1mg/ml PGRN/GelMA complex from week 0 for 4 weeks (dose of 50µL/tooth). | Three groups:  Control Group (n=8 teeth from n=8 dogs): no intervention;  SRP Group (n=8 teeth from n=8 dogs): SRP once only at week 0;  SRP + GelMA Group (n=8 teeth from n=8 dogs): SRP once followed by weekly sub-gingival delivery of 5% w/v GelMA from week 0 for 4 weeks. | *In vivo* (Dog model).  Population: 12-to-14-month-old male Beagle dogs, N=8 dogs, N=32 teeth (PC).  Duration: experiment end 8 weeks after initiating treatment.  Protocol: randomised/blinded allocation not reported. Blinded examiner. Class II furcation defects were surgically created at mandibular teeth at baseline. Infection- and ligature-induced periodontitis (*P.g.* applied to furcation defects then the roots of mandibular third and fourth premolars were ligated). | Alveolar bone quantity in furcation defect using radiographic parameters (BV/TV, trabecular thickness, Tb.Sp) and histological parameters (percentage of new bone area [NBA%], cementum length [NCL%], epithelium length [EL%]); bleeding index (BI); PPD; gingival recession depth (GRD);  AL; protein expression levels of M1 macrophage-related marker (TNF-α), M2 macrophage-related marker (IL-10), Th17 cell-related marker (IL-17) and Treg cell-related marker (IL-35) in GCF; gene expression of M1 macrophage-related markers (*TNF-α, iNOS*), Th17 cell-related markers (*RANKL, RORγt*), M2 macrophage-related markers (*CD206, TGF-β, IL-10*), Treg cell-related markers (*Foxp3*) and the anti-inflammatory marker *OPG* | Lost at baseline: n=1.  At baseline, no difference in BI, PPD, GRD and AL between the Groups  At week 8:   - Lower BI, PPD and AL than all controls, and lower GRD compared to Control Group - Reduced gene expression of *TNF-α, iNOS, RANKL* & *RORγt* and reduced protein expression of TNF-α and IL-17 compared to all controls - Higher gene expression of *CD206, TGF-β, IL-10, OPG* & *Foxp3* and higher protein expression of IL-10 & IL-35 compared to all controls - Greater BV/TV, trabecular thickness, NBA% and NCL% compared to all controls - Reduced Tb.Sp compared to all controls. Lower EL% than the Control and SRP Groups   At week 8, visibly greater alveolar bone filling of furcation defect than SRP and SRP + GelMA Groups radiographically and histologically (no *p*-values) |
| *Combination of agents (co-administration of ≥2 types of agents)* | | | | | |
| Llavaneras *et al.* (2001) | Three groups with periodontitis:  CMT-8 Group (n=6): daily oral gavage, 1mg chemically modified tetracycline-(CMT-)8 in 1ml CMC;  Clodronate Group (n=6): weekly subcutaneous injections, 1mg clodronate (a bisphosphonate) in 1ml saline;  Combination Group (n=6): daily oral gavage, 1mg CMT-8 in 1ml CMC, and weekly subcutaneous injection of 1mg clodronate in 1ml saline. | Two groups:  Control Group (n=6): without periodontitis, daily oral gavage with 1ml CMC;  LPS Group (n=6): with periodontitis, daily oral gavage with 1ml CMC. | *In vivo* (Rat model).  Population: adult male Sprague-Dawley rats, N=30 (NPC).  Duration: Experiment end at day 7.  Protocol: randomised/blinding allocation not reported. Examiner blinding not reported. Infection-induced periodontitis (*E.c.* and LPS). | Tooth mobility score for molar teeth; alveolar bone loss (ACJ-ABC distance); collagenase, gelatinase and elastase activity; MMP-2, MMP-8, MMP-9 and MMP-13 expression | Combination Group had reduced tooth mobility score and alveolar bone loss compared to LPS Group.  Visually reduced collagenase, gelatinase and elastase activity in Clodronate and CMT-8 Groups. Markedly reduced in Combination Group with inhibition of both inactive and active forms of collagenases (limited to active collagenases for other intervention groups) (no *p*-values).  Reduced MMP-2, MMP-8 and MMP-9 expression in the CMT-8, Clodronate and Combination Groups compared to LPS Group (no *p*-values). |
| *^a^* All findings reported are statistically significant unless otherwise stated.  *Abbreviations:*  Sample size calculation or power analysis not reported (NPC), sample size calculation or power analysis reported (PC);  Bacteria: *Aggregatibacter actinomycetemcomitans (A.a.)*, *Escherichia coli* *(E.c.)*, *Fusobacterium nucleatum (F.n.)*, *Porphyromonas gingivalis (P.g.)*, *Prevotella intermedia (P.i.), Treponema denticola (T.d.)*, *Tannerella forsythia (T.f.)*;  Alveolar bone crest (ABC), American Academy of Periodontology (AAP), amelo-cemental junction (ACJ), attachment loss (AL), Basal Fibroblast Medium (BFM), bleeding index (BI), bone marrow macrophages (BMMs), bone marrow stromal cell (BMSC), bone volume/tissue volume (BV/TV), carboxymethylcellulose (CMC), cathepsin K (CTSK), cervical lymph node (CLN), cyclooxygenase (COX), extracellular matrix (ECM), formalin-killed (FK), forkhead box P3 (Foxp3), gingival crevicular fluid (GCF), gingival index (GI), gingival recession depth (GRD), immunoglobulin (Ig), inducible nitric oxide synthase (iNOS), interferon (IFN), interleukin (IL), lipopolysaccharide (LPS), macrophage colony-stimulating factor (M-CSF), mammalian target of rapamycin (mTOR), matrix metalloproteinase (MMP), modified gingival index (MGI), osteoprotegerin (OPG), oral hygiene instruction (OHI), percentage of epithelium length (EL%), percentage of new bone area (NBA%), percentage of new cementum length (NCL%), phosphate-buffered saline (PBS), plaque index (PI), probing pocket depth (PPD), prostaglandin E2 (PGE2), reactive oxygen species (ROS), receptor activator of nuclear factor kappa-B ligand (RANKL), retinoic acid-related orphan receptor gamma t (RORγt), scaling and root planing (SRP), submandibular lymph node (SMLN), tartrate-resistant acid phosphatase (TRAP), tissue inhibitor of metalloproteinase (TIMP), transforming growth factor (TGF), trabecular separation (Tb.Sp), tumor necrosis factor (TNF), 3-nitrotyrosine (3NT). | | | | | |

References

1.	Caton JG, Ciancio SG, Blieden TM, Bradshaw M, Crout RJ, Hefti AF, et al. Treatment with subantimicrobial dose doxycycline improves the efficacy of scaling and root planing in patients with adult periodontitis. Journal of Periodontology. 2000;71(4):521-32.

2.	Caton JG, Ciancio SG, Blieden TM, Bradshaw M, Crout RJ, Hefti AF, et al. Subantimicrobial dose doxycycline as an adjunct to scaling and root planing: post-treatment effects. Journal of Clinical Periodontology. 2002;28(8):782-9.

3.	Choi D, Moon I, Choi B, Paik J, Kim Y, Choi S, et al. Effects of sub-antimicrobial dose doxycycline therapy on crevicular fluid MMP-8, and gingival tissue MMP-9, TIMP-1 and IL-6 levels in chronic periodontitis. Journal of periodontal research. 2004;39(1):20-6.

4.	Emingil G, Atilla G, Sorsa T, Luoto H, Kirilmaz L, Baylas H. The Effect of Adjunctive Low-Dose Doxycycline Therapy on Clinical Parameters and Gingival Crevicular Fluid Matrix Metalloproteinase-8 Levels in Chronic Periodontitis. Journal of periodontology. 2004;75(1):106-15.

5.	Gilowski L, Kondzielnik P, Wiench R, Plocica I, Strojek K, FKrzeminski TF. Efficacy of short-term adjunctive subantimicrobial dose doxycycline in diabetic patients – randomized study. Oral diseases. 2012;18(8):763-70.

6.	Golub LM, Ciancio S, Ramamurthy NS, Leung M, McNamara TF. Low-dose doxycycline therapy: Effect on gingival and crevicular fluid collagenase activity in humans. Journal of Periodontal Research. 1990;25(6):321-30.

7.	Golub LM, McNamara TF, Ryan ME, Kohut B, Blieden T, Payonk G, et al. Adjunctive treatment with subantimicrobial doses of doxycycline: effects on gingival fluid collagenase activity and attachment loss in adult periodontitis. Journal of Clinical Periodontology. 2001;28(2):146-56.

8.	Needleman I, Suvan J, Gilthorpe MS, Tucker R, George GS, Giannobile W, et al. A randomized-controlled trial of low-dose doxycycline for periodontitis in smokers. Journal of Clinical Periodontology. 2007;34(4):325-33.

9.	Parvu AE, Alb SF, Craciun A, Taulescu MA. Efficacy of subantimicrobial-dose doxycycline against nitrosative stress in chronic periodontitis. Acta Pharmacologica Sinica. 2013;34:247-54.

10.	Preshaw PM, Hefti AF, Novak MJ, Michalowicz BS, Pihlstrom BL, Schoor R, et al. Subantimicrobial dose doxycycline enhances the efficacy of scaling and root planing in chronic periodontitis: a multicenter trial. Journal of periodontology. 2004;75(8):1068-76.

11.	Reinhardt RA, Stoner JA, Golub LM, Wolff MS, Lee H, Meinberg TA, et al. Efficacy of sub-antimicrobial dose doxycycline in post-menopausal women: clinical outcomes. Journal of Clinical Periodontology. 2007;34(9):768-75.

12.	Branco-de-Almeida LS, Franco GC, Castro ML, dos Santos JG, Anbinder AL, Cortelli SC, et al. Fluoxetine Inhibits Inflammatory Response and Bone Loss in a Rat Model of Ligature-Induced Periodontitis. Journal of Periodontology. 2012;83(5):664-71.

13.	Coat J, Demoersman J, Beuzit S, Cornec D, Devauchelle-Pensec V, Saraux A, et al. Anti-B lymphocyte immunotherapy is associated with improvement of periodontal status in subjects with rheumatoid arthritis. Journal of clinical periodontology. 2015;42(9):817-23.

14.	Grauballe MB, Ostergaard JA, Schou S, Flyvbjerg A, Holmstrup P. Effects of TNF-α blocking on experimental periodontitis and type 2 diabetes in obese diabetic Zucker rats. Journal of Clinical Periodontology. 2015;42(9):807-16.

15.	Hao L, Chen J, Zhu Z, Reddy MS, Mountz JD, Chen W, et al. Odanacatib, A Cathepsin K-Specific Inhibitor, Inhibits Inflammation and Bone Loss Caused by Periodontal Diseases. Journal of Periodontology. 2015;86(8):972-83.

16.	Kobayashi T, Okada M, Ito S, Kobayashi D, Ishida K, Kojima A, et al. Assessment of interleukin-6 receptor inhibition therapy on periodontal condition in patients with rheumatoid arthritis and chronic periodontitis. Journal of periodontology. 2014;85(1):57-67.

17.	Kobayashi T, Yokoyama T, Ito S, Kobayashi D, Yamagata A, Okada M, et al. Periodontal and serum protein profiles in patients with rheumatoid arthritis treated with tumor necrosis factor inhibitor adalimumab. Journal of periodontology. 2014;85(11):1480-8.

18.	Mayer Y, Balbir-Gurman A, Machtei EE. Anti-tumor necrosis factor-alpha therapy and periodontal parameters in patients with rheumatoid arthritis. Journal of periodontology. 2009;80(9):1414-20.

19.	Mayer Y, Elimelech R, Balbir-Gurman A, Braun-Moscovici Y, Machtei EE. Periodontal condition of patients with autoimmune diseases and the effect of anti-tumor necrosis factor-α therapy. Journal of periodontology. 2013;84(2):136-42.

20.	Napimoga MH, Rocha EP, Trindade-da-Silva CA, Demasi APD, Martinez EF, Macedo CG, et al. Soluble epoxide hydrolase inhibitor promotes immunomodulation to inhibit bone resorption. Journal of periodontal research. 2018;53(5):743-9.

21.	Pers JO, Saraux A, Pierre R, Youinou P. Anti-TNF-alpha immunotherapy is associated with increased gingival inflammation without clinical attachment loss in subjects with rheumatoid arthritis. Journal of Periodontology. 2008;79(9):1645-51.

22.	Vieira GHA, Rivas ACA, Costa KF, Oliviera LFF, Suzuki KT, Messora MR, et al. Specific inhibition of IL-6 receptor attenuates inflammatory bone loss in experimental periodontitis. Journal of Periodontology. 2021;92(10):1460-9.

23.	O'Brien-Simpson NM, Holden JA, Lenzo JC, Tan Y, Brammar GC, Walsh KA, et al. A therapeutic Porphyromonas gingivalis gingipain vaccine induces neutralising IgG1 antibodies that protect against experimental periodontitis. npj Vaccines. 2016;1.

24.	Sugano N, Takane M, Yoshinuma N, Ito K. Adjunctive effects of anti-Porphyromonas gingivalis egg yolk antibody with scaling and root planning: a randomized, placebo-controlled clinical trial. Journal of Periodontology. 2009;80(11):1901-3.

25.	Abidi AH, Abhyankar V, Alghamdi SS, Tipton DA, Dabbous M. Phytocannabinoids regulate inflammation in IL-1beta-stimulated human gingival fibroblasts. Journal of periodontal research. 2022.

26.	Cafferata EA, Castro-Saavedra S, Fuentes-Barros G, Melgar-Rodriguez S, Rivera F, Carvajal P, et al. Boldine inhibits the alveolar bone resorption during ligature-induced periodontitis by modulating the Th17/Treg imbalance. Journal of Periodontology. 2021;92(1):123-36.

27.	Kim Y, Kim MO, Kim S, Kim HJ, Pokhrel NK, Lee JH, et al. 6-Shogaol, an active ingredient of ginger, inhibits osteoclastogenesis and alveolar bone resorption in ligature-induced periodontitis in mice. Journal of Periodontology. 2019;91(6):809-18.

28.	Leguizamon NDP, de Molon RS, Coletto-Nunes G, Nogueira AVB, Rocha SV, Neo-Justino DM, et al. Phytocystatin CsinCPI-2 Reduces Osteoclastogenesis and Alveolar Bone Loss. Journal of dental research. 2022;101(2):216-25.

29.	Kawahara Y, Kaneko T, Yoshinaga Y, Arita Y, Nakamura K, Koga C, et al. Effects of Sulfonylureas on Periodontopathic Bacteria-Induced Inflammation. Journal of dental research. 2020;99(7):830-8.

30.	Zhou X, Zhang P, Wang Q, Ji N, Xia S, Ding Y, et al. Metformin ameliorates experimental diabetic periodontitis independently of mammalian target of rapamycin (mTOR) inhibition by reducing NIMA-related kinase 7 (Nek7) expression. Journal of Periodontology. 2019;90(9):1032-42.

31.	Tsutsumi K, Fujikawa H, Kajikawa T, Takedachi M, Yamamoto T, Murakami S. Effects of L-ascorbic acid 2-phosphate magnesium salt on the properties of human gingival fibroblasts. Journal of periodontal research. 2011;47(2):263-71.

32.	Wang L, Wang J, Jin Y, Gao H, Lin X. Oral administration of all-trans retinoic acid suppresses experimental periodontitis by modulating the Th17/Treg imbalance. Journal of periodontology. 2014;85(5):740-50.

33.	Yu H, Valerio M, Bielawski J. Fenretinide inhibited de novo ceramide synthesis and proinflammatory cytokines induced by Aggregatibacter actinomycetemcomitans. Journal of Lipid Research. 2013;54(1):189-201.

34.	Glowacki AJ, Yoshizawa S, Jhunjhunwala S, Little SR. Prevention of inflammation-mediated bone loss in murine and canine periodontal disease via recruitment of regulatory lymphocytes. Proceedings of the National Academy of Sciences of the United States of America (PNAS). 2013;110(46):18525-30.

35.	He Z, Liu Y, Zheng ZL, Lv JC, Liu SB, Zhang J, et al. Periodic Lamellae-Based Nanofibers for Precise Immunomodulation to Treat Inflammatory Bone Loss in Periodontitis. Advanced Healthcare Materials. 2024:e2303549.

36.	Branco-de-Almeida LS, Franco GCN, Castro ML, Vieira MS, Galvao-Moreira LV, Cortelli SC, et al. Protective effects of desipramine on alveolar bone in experimental periodontitis. Journal of Periodontology. 2020;91(12):1694-703.

37.	Breivik T, Gundersen Y, Myhrer T, Fonnum F, Osmundsen H, Murison R, et al. Enhanced susceptibility to periodontitis in an animal model of depression: reversed by chronic treatment with the anti-depressant tianeptine. Journal of Clinical Periodontology. 2006;33(7):469-77.

38.	Luo H, Chen D, Li R, Li R, Teng Y, Cao Y, et al. Genetically engineered CXCR4-modified exosomes for delivery of miR-126 mimics to macrophages alleviate periodontitis. Journal of Nanobiotechnology. 2023;21(1):116.

39.	Breivik T, Opstad PK, Engstad R, Gundersen G, Gjermo P, Preus H. Soluble beta-1,3/1,6-glucan from yeast inhibits experimental periodontal disease in Wistar rats. Journal of Clinical Periodontology. 2005;32(4):347-52.

40.	Karakan NC, Akpinar A, Goze F, Poyraz O. Investigating the Effects of Systemically Administered Strontium Ranelate on Alveolar Bone Loss Histomorphometrically and Histopathologically on Experimental Periodontitis in Rats. Journal of Periodontology. 2017;88(2):e24-e31.

41.	Liu W, Song A, Wu Y, Gong P, Zhao J, Zhang L, et al. Enhanced immunomodulation and periodontal regeneration efficacy of subgingivally delivered progranulin-loaded hydrogel as an adjunct to non-surgical treatment for Class II furcation involvement in dogs. Journal of Clinical Periodontology. 2024;51(6):774-86.

42.	Llavaneras A, Ramamurthy NS, Heikkila P, Teronen O, Salo T, Rifkin BR, et al. A combination of a chemically modified doxycycline and a bisphosphonate synergistically inhibits endotoxin-induced periodontal breakdown in rats. Journal of periodontology. 2001;72(8):1069-77.
